# Temporal‐Targeted Accelerated 40 Hz tACS Enhances Cognitive Function and Network Integration in Alzheimer's Disease: A Randomized Clinical Trial

**DOI:** 10.1002/advs.77851

**Published:** 2026-09-27

**Authors:** Rong Guo, Xingxing Li, Chenjun Zou, Chao Zheng, Xiaohong Wu, Xuechao Lu, Jiaying Yan, Junfang Zhang, Xiaoyan Luo, Shiwei Ye, Dongsheng Zhou

**Affiliations:** ^1^ Department of Psychiatry Ningbo Key Laboratory for Physical Diagnosis and Treatment of Mental and Psychological Disorders Affiliated Kangning Hospital of Ningbo University (Ningbo Kangning Hospital) Ningbo Zhejiang People's Republic of China; ^2^ School of Basic Medical Sciences Health Science Center Ningbo University Ningbo People's Republic of China; ^3^ Department of Psychiatry The Second People's Hospital of Lishui Lishui Zhejiang People's Republic of China

**Keywords:** 40 Hz transcranial alternating current stimulation, alzheimer's disease, cognitive function, functional connectivity, functional near‐infrared spectroscopy

## Abstract

The temporal lobes are key hubs for memory and cognition. Although 40 Hz transcranial alternating current stimulation (tACS) has shown potential cognitive benefits, evidence for accelerated bilateral temporal stimulation remains limited. This study aims to evaluate its cognitive and neural effects. In this randomized, double‐blind, controlled trial, 100 Alzheimer's disease (AD) patients were randomized 1:1 to active or sham 40 Hz tACS over the bilateral temporal. Cognitive outcomes at three time points were analyzed using mixed repeated‐measures analysis of variance (ANOVA). Resting‐state functional near‐infrared spectroscopy (fNIRS) data from baseline and Week 4 were analyzed using ANOVA, followed by network‐based statistics with family‐wise error (FWE) correction. Eighty‐five participants completed the trial. Significant Group × Time interactions were observed for Alzheimer's Disease Assessment Scale‐Cognitive Subscale (ADAS‐Cog) total (*p* = 0.010), memory (*p* = 0.007), and praxis (*p* = 0.002), but not for the Mini‐Mental State Examination (MMSE) (*p* = 0.075). The fNIRS analysis revealed a functional network primarily involving the frontal and parietal regions, comprising 38 connections and 18 nodes (*p_FWE_* = 0.010). Active‐group connectivity changes were associated with ADAS‐Cog total, language, praxis, and MMSE variations, but not in the sham group. Accelerated bilateral temporal 40 Hz tACS improved cognition and modulated frontotemporoparietal connectivity. Larger multicenter studies with biomarkers are warranted.

## Graphical abstract

Bilateral temporal accelerated 40‐Hz tACS improves cognitive function in patients with Alzheimer's disease. Resting‐state fNIRS identifies treatment‐related increases in frontotemporoparietal functional connectivity, which are associated with cognitive improvement.

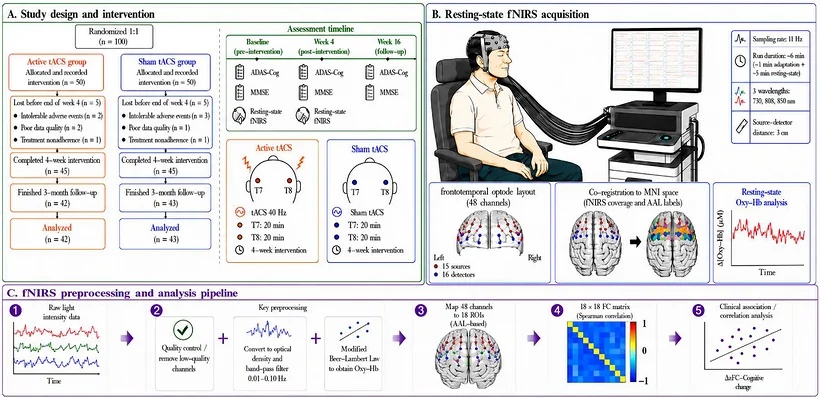

## 1 Introduction

Alzheimer's disease (AD) is the most common form of dementia and is characterized by progressive impairments in memory, language, and executive function, imposing a substantial burden on patients, families, and society [1]. Current pharmacological treatments may alleviate symptoms or slow cognitive and functional decline in selected patients; however, their overall clinical benefits remain limited, and their application may be constrained by disease stage, patient eligibility, safety‐monitoring requirements, treatment delivery, and healthcare resources [2]. Therefore, the development of safe, well‐tolerated, and repeatable non‐pharmacological interventions remains an important priority in AD treatment. Non‐invasive brain stimulation provides a potential therapeutic approach by modulating abnormal neural activity associated with cognitive dysfunction in AD. Among these techniques, transcranial electrical stimulation has attracted increasing attention because of its relatively simple operation, compact equipment, and suitability for repeated administration, particularly transcranial alternating current stimulation (tACS). Although preliminary studies with relatively small samples have suggested potential cognitive benefits, the clinical efficacy and underlying mechanisms of tACS require confirmation in larger randomized controlled trials.

Transcranial alternating current stimulation applies sinusoidal electrical currents at predefined frequencies to modulate endogenous neural oscillations, thereby providing a potential means of targeting abnormal brain rhythms in AD. Gamma oscillations are involved in attentional processing, memory encoding, and the coordination of information across brain regions [3], whereas abnormalities in gamma‐band activity and cross‐frequency coupling have been reported in patients with AD [4]. The 40 Hz gamma oscillation plays a pivotal role in improving AD‐related cognitive functions. Animal studies have shown that 40‐Hz rhythmic stimulation may regulate microglial responses, reduce amyloid‐β (Aβ) and phosphorylated tau pathology, attenuate neuronal and synaptic damage, and improve learning and memory performance [5, 6]. An open‐label study using repeated 40‐Hz tACS predominantly targeting the temporal lobes reported increased temporal perfusion, with perfusion changes being associated with gamma activity and selected memory outcomes [7]. A randomized controlled trial targeting the dorsolateral prefrontal cortex (DLPFC) demonstrated improvements in global cognition, accompanied by increased frontocentral theta power, enhanced hippocampal–prefrontal theta‐band connectivity, and increased local low‐gamma connectivity [8]. Another randomized controlled trial employing an accelerated, high‐intensity frontomastoid tACS protocol in patients with mild AD reported cognitive improvement together with increased functional connectivity (FC) within cortical networks and between the hippocampus and specific cortical regions [9]. Collectively, these findings suggest that the effects of 40‐Hz tACS may extend beyond local modulation of neural oscillations and involve alterations in large‐scale functional brain networks.

The primary pathogenic mechanisms of AD currently involve amyloid‐β (Aβ) deposition and tau protein abnormalities, while AD is also characterized by impaired integration of large‐scale brain networks [10, 11]. Abnormalities in long‐range FC involve widespread frontal, temporal, and parietal regions, and reduced network connectivity has been associated with poorer global cognitive function [12]. Examining the effects of 40‐Hz tACS at the network level may therefore improve our understanding of its potential neural mechanisms. Non‐invasive methods commonly used to assess FC include functional magnetic resonance imaging (fMRI), electroencephalography (EEG), and functional near‐infrared spectroscopy (fNIRS). Functional near‐infrared spectroscopy measures spontaneous fluctuations in cortical oxygenated and deoxygenated hemoglobin signals and can be used to estimate resting‐state FC between cortical regions covered by the optode array. It is non‐invasive, portable, relatively tolerant of movement, and suitable for repeated assessments, making it particularly applicable to older adults and individuals with cognitive impairment [13, 14]. Resting‐state fNIRS may therefore provide a feasible approach for dynamically assessing intervention‐related changes in frontotemporoparietal FC and for investigating the network‐level effects of 40‐Hz tACS.

Within this distributed network, the temporal lobe is a key node for memory and higher‐order cognition and is among the regions affected relatively early in Alzheimer's disease [15]. Based on previous findings showing favorable changes in temporal and hippocampal perfusion following temporal‐targeted stimulation, the present study selected T7 and T8, defined according to the international 10–20 electroencephalographic system, as standardized scalp locations for bilateral temporal stimulation [7]. A 2‐mA intensity was chosen, as it has been widely used in previous studies with demonstrated feasibility and tolerability [16]. Given that the neuromodulatory after‐effects of a single tACS session may be relatively transient, increasing the number of daily sessions may enhance cumulative effects; preliminary evidence suggests that twice‐daily 40 Hz tACS may yield greater symptom improvement than once‐daily stimulation [17]. However, the cumulative effects of repeated stimulation may depend on the intersession interval and the state of neural plasticity, as excessively short intervals may attenuate or even reverse the after‐effects of the preceding session [18]. Previous clinical studies have demonstrated the feasibility of administering two daily transcranial electrical stimulation sessions separated by several hours [9, 19]. Accordingly, considering patient schedules during hospitalization, the intersession interval in the present study was set at a minimum of 4 h to balance treatment intensity with the preservation of stimulation after‐effects.

Against this background, we conducted a prospective, randomized, double‐blind, sham‐controlled trial to evaluate a 4‐week course of bilateral temporal 40‐Hz tACS in patients with AD. Stimulation was delivered over T7 and T8 twice daily for 20 min per session, with an interval of at least 4 h between sessions. Cognitive function was assessed at baseline, Week 4, and Week 16 using the Alzheimer's Disease Assessment Scale–Cognitive Subscale (ADAS‐Cog) and the Mini‐Mental State Examination (MMSE). Resting‐state fNIRS data were acquired before and after the intervention to evaluate changes in FC within the frontotemporoparietal network (FTPN) and to examine the associations between network‐level changes and changes in cognitive outcomes.

## 2 Methods

### 2.1 Study Design

This clinical trial was registered in the Chinese Clinical Trial Registry (ChiCTR2300068381) before participant enrollment at http://www.chictr.org.cn. The study was approved by the Ethics Committee of Affiliated Kangning Hospital of Ningbo University. All procedures were conducted in accordance with the Declaration of Helsinki. Written informed consent was obtained from all participants or their legally authorized representatives before any study procedure.

A total of 253 individuals were screened for eligibility. Of these, 153 were excluded: 97 did not meet the inclusion criteria, and 56 declined to participate. The remaining 100 patients were randomly assigned in a 1:1 ratio to either the active group (*n* = 50) or the sham group (*n* = 50). Before completion of the 4‐week intervention, five participants in each group withdrew. Reasons for withdrawal in the active group included intolerable adverse events (*n* = 2), poor data quality (*n* = 2), and treatment nonadherence (*n* = 1). In the sham group, withdrawals were attributed to intolerable adverse events (*n* = 3), poor data quality (*n* = 1), and treatment nonadherence (*n* = 1). Consequently, 45 participants in each group completed the full 4‐week intervention. During follow‐up, three additional participants in the active group and two in the sham group were lost to follow‐up. At Week 16, 42 participants remained in the active group and 43 in the sham group. These 85 participants constituted the final analysis sample. Allocation concealment was implemented using sequentially numbered, opaque, sealed envelopes prepared by a researcher who was not involved in participant recruitment, outcome assessment, or stimulation delivery.

**FIGURE 1:**
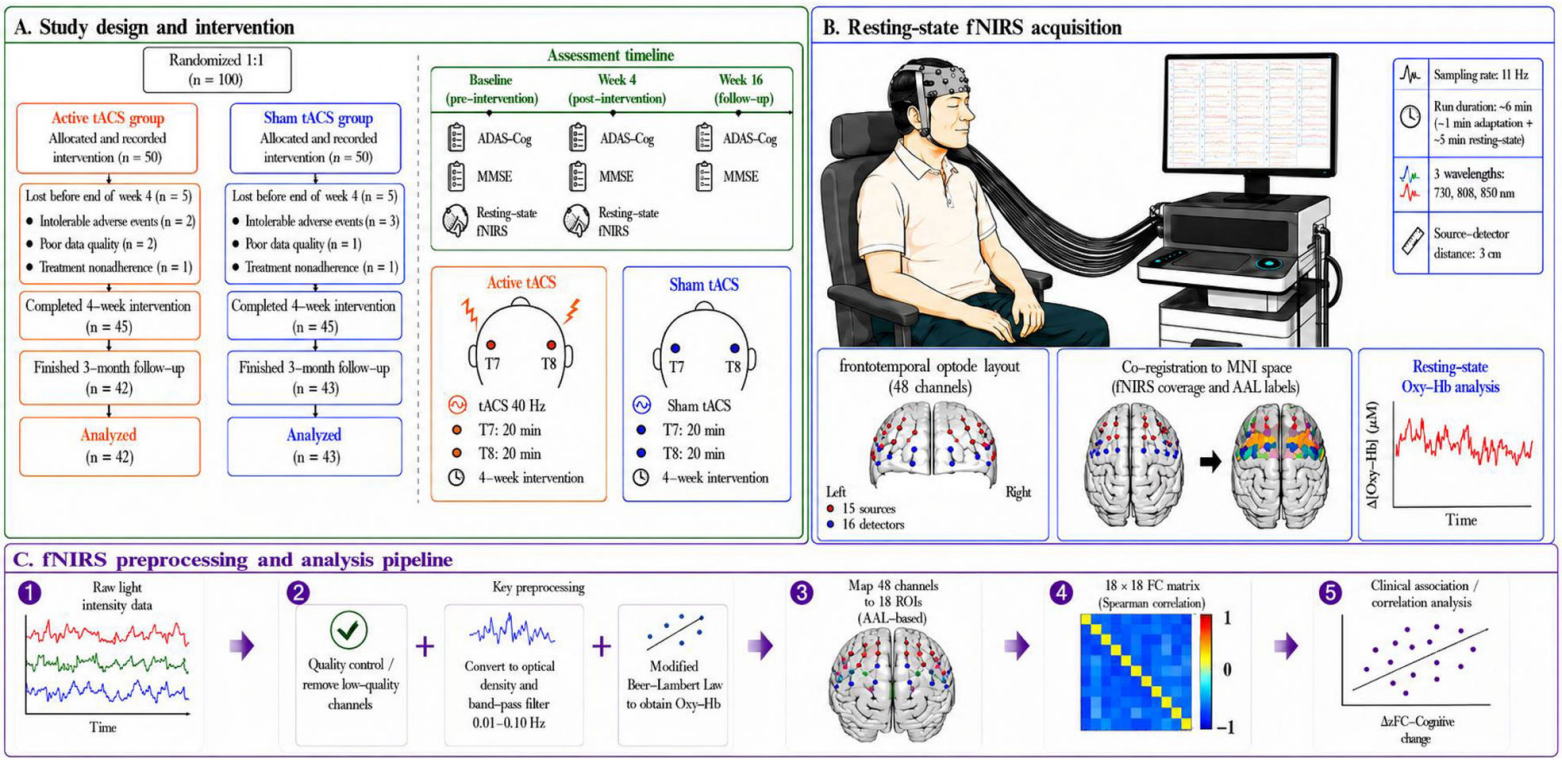
Study design, resting‐state fNIRS acquisition, and analysis pipeline. (A) Overview of the randomized controlled trial design and intervention protocol. (B) Resting‐state fNIRS acquisition procedure, cap layout, recording parameters, optode placement, and 3D co‐registration to MNI space and AAL labels. (C) fNIRS preprocessing and analysis pipeline, including raw light‐intensity data, quality control, conversion to optical density, band‐pass filtering, conversion to Oxy‐Hb using the modified Beer‐Lambert law, ROI‐level mapping, FC matrix construction, and clinical association analysis. **Abbreviations**: AAL, Automated Anatomical Labeling; ADAS‐Cog, Alzheimer's Disease Assessment Scale–Cognitive Subscale; FC, functional connectivity; fNIRS, functional near‐infrared spectroscopy; MNI, Montreal Neurological Institute; MMSE, Mini‐Mental State Examination; Oxy‐Hb, oxygenated hemoglobin; ROI, region of interest; tACS, transcranial alternating current stimulation.

The study included a 4‐week tACS intervention period (hereafter referred to as Week 4) and a 3‐month follow‐up period without tACS intervention (hereafter referred to as Week 16). All researchers and participants were blinded to the intervention group allocation. The study flow is illustrated in Figure 1.

### 2.2 Participants

Participants were recruited from Ningbo Kangning Hospital from February 2023 to July 2025, with the inclusion criteria: (1) meeting the diagnostic criteria for AD according to the *International Classification of Diseases, 10th Revision (ICD‐10)*, (2) age ≥ 55 years, (3) right‐handed, (4) use of the same cholinesterase inhibitor, donepezil (the dose was fixed and not adjusted during the study period), and (5) written informed consent from the participant or legal guardian.

Exclusion criteria included: (1) patients with severe physical illnesses, infectious diseases, or immune system disorders; (2) patients with severe neurological diseases, intellectual disability, or organic brain disorders; (3) patients with other mental illnesses, including depression, schizophrenia, and delirium; (4) history of epileptic seizures; (5) receipt of electroconvulsive therapy (ECT) within the past 3 months; and (6) clinical unsuitability for tACS or inability to complete the required assessments.

### 2.3 Intervention Measures

Participants in the active tACS group received bilateral temporal lobe tACS, targeting the T7 and T8 electrode sites as defined by the International 10–20 EEG system. Saline‐soaked sponges (size: 4 × 4 cm^2^) were used to deliver the stimulation via a transcranial stimulator (Kerfun Medical Co., Ltd., Xi'an, China). A 40 Hz sinusoidal current was applied at an intensity of 2 mA for 20 min per session. The intervention was administered twice daily, 5 days a week, with an interval of at least 4 h between the two daily sessions, for a total of 40 sessions over four consecutive weeks.

Participants in the sham stimulation group used the same tACS stimulator but received a sham stimulation paradigm: the current rose to 2 mA within 3 s at the start, maintained this current for 30 s, and then gradually decreased to 0 mA, without exerting any stimulatory effect [20]. The sham group followed the same session schedule (twice daily, 5 days per week, for 4 weeks) as the active group.

### 2.4 Cognitive Assessment

Two neurologists (each with >10 years of clinical experience in dementia) were trained to administer the scales according to standardized procedures and were blinded to treatment allocation. The ADAS‐Cog consists of 11 items, which are categorized into four subdomains: language, memory, praxis, and attention. The total score ranges from 0 to 70, with higher scores indicating more severe cognitive impairment. The total score of the MMSE ranges from 0 to 30, with higher scores reflecting better cognitive function.

Before study initiation, the assessors underwent standardized training. An intraclass correlation coefficient of at least 0.80 was required to establish adequate inter‐rater reliability. Discrepancies were resolved by discussion, and additional cases were independently reassessed until the prespecified criterion was met for each scale. Cognitive assessments were conducted at baseline (within 24 h before the intervention), Week 4 (the next day after completion of the intervention), and Week 16 (at the end of follow‐up). The primary outcome was the longitudinal change in ADAS‐Cog total score across the three assessment time points. Secondary outcomes were longitudinal changes in ADAS‐Cog domain (language, memory, praxis, attention) scores and the MMSE total score.

### 2.5 fNIRS Acquisition and Functional Connectivity Analysis

Resting‐state cerebral hemodynamic activity was recorded with a 48‐channel multi‐channel NIRS system (NirScan‐4000B, Danyang Huichuang Medical Equipment Co., Ltd., China; sampling rate 11 Hz). Recordings were obtained in a dim, quiet room while participants sat comfortably with eyes closed, refraining from talking or large movements. Each run lasted ∼6 min (1‐min adaptation plus 5‐min resting state). The cap contained 15 sources and 16 detectors arranged over the frontal and bilateral temporal cortices (Figure 1), with three wavelengths (730, 808, 850 nm) and a source–detector distance of 3 cm. Optodes were positioned according to the international 10–20 EEG system, with detector D3 at FPz and the midline aligned to the FPz–Oz axis. Three‐dimensional co‐registration based on the manufacturer's cap configuration was used to derive Montreal Neurological Institute (MNI) coordinates and corresponding Automated Anatomical Labeling (AAL) regions for each channel (Table S1). Resting‐state fNIRS data were collected at baseline (24 h before the first stimulation) and at Week 4, which is the day after the last stimulation session.

The fNIRS preprocessing and FC analyses were performed in NirSpark. Saturated or low‐quality channels were discarded, raw light‐intensity data were converted to optical density (OD), and a 0.01–0.10 Hz band‐pass filter was applied to remove slow drift and physiological noise. OD signals were then transformed to changes in oxygenated hemoglobin (Oxy‐Hb) using the modified Beer–Lambert law. The 48 channels were mapped to 18 cortical regions of interest (ROIs) based on the AAL template, and channels within each ROI were averaged to obtain ROI‐level time series. Pearson correlations between all ROI pairs yielded 18 × 18 symmetric FC matrices. For each valid connection, a 2‐group (active vs. sham) × 2‐time‐point (baseline vs. Week 4) mixed repeated‐measures analysis of variance was performed, with the Group × Time interaction as the effect of interest. Network‐based statistic (NBS) was used to identify treatment‐related network changes while controlling for multiple comparisons across ROI‐to‐ROI connections. Connections with an uncorrected interaction *p* < 0.05 formed the initial edge‐level threshold, and suprathreshold edges sharing nodes were grouped into connected components. Component‐level significance was assessed using 5000 nonparametric permutations. For each permutation, group labels were randomly reassigned, and the number of edges in the largest component was recorded to generate the empirical null distribution. Components with family‐wise error‐corrected *p* (*p_FWE_*) < 0.05 were considered significant. Between‐group differences in mean NBS‐derived delta Fisher z‐transformed FC (ΔzFC) were explored using Welch's independent‐samples *t*‐test.

### 2.6 Brain–Behavior Correlation Analysis

For each participant, mean ΔzFC was calculated by averaging the Week 4 minus baseline connectivity change across all edges in the significant NBS component:

ΔzFC=zFCWeek4−zFCBaseline

Changes in the six cognitive outcomes were calculated in the same direction. Spearman correlation analyses were performed separately in the active and sham groups to examine associations between mean ΔzFC and changes in ADAS‐Cog total, language, memory, praxis, attention, and MMSE total scores. The p‐value has been corrected using the false discovery rate (FDR).

### 2.7 Safety and Tolerability Assessment

During the intervention, safety was monitored using a structured adverse event (AE) checklist completed after each tACS session. Participants and their caregivers were instructed to report any new or worsening symptoms promptly. All AEs were coded according to the Common Terminology Criteria for Adverse Events (CTCAE), version 5.0, and were followed until resolution. Serious adverse events (SAEs) triggered immediate clinical management, tACS adjustment/discontinuation, or study withdrawal with continued medical follow‐up. The incidence rate of adverse events was calculated for each group.

### 2.8 Sample Size Calculation

The sample size was calculated to detect a Group × Time interaction in the ADAS‐Cog total score. A small‐to‐moderate effect size (f = 0.20) was assumed [21, 22], with 95% power, a two‐sided α of 0.05, two groups, three repeated measurements, and a correlation of 0.50 among repeated measures. G*Power version 3.1 estimated a minimum total sample of 66 participants.

### 2.9 Statistical Analysis

Baseline demographic and clinical characteristics were compared between groups using independent‐samples t tests or Mann–Whitney U tests for continuous variables, as appropriate, and χ^2^ tests or Fisher's exact tests for categorical variables. Cognitive outcomes were analyzed using 2‐group × 3‐time‐point mixed repeated‐measures analyses of variance, with Group (active vs. sham) as the between‐subject factor and Time (baseline, Week 4, and Week 16) as the within‐subject factor. Sphericity was assessed using Mauchly's test. When sphericity was satisfied, sphericity‐assumed results were reported; when it was violated, Greenhouse–Geisser‐corrected degrees of freedom and p values were reported. Omnibus *p*‐values for Group, Time, and Group × Time effects were unadjusted. Significant interactions were followed by simple‐effects analyses and Bonferroni‐adjusted pairwise comparisons. Change scores from baseline to Week 4 and Week 16 were additionally compared between groups using Welch's independent‐samples t tests, with FDR correction. Mean zFC across all edges in the significant NBS component was analyzed using a 2‐group × 2‐time‐point mixed repeated‐measures analysis of variance. Subsequent simple‐effects and pairwise‐comparison *p*‐values were Bonferroni‐adjusted. Effect sizes are reported as partial eta squared (ηp^2^) for analyses of variance and Cohen's d or dz for independent or paired comparisons, respectively. Mixed repeated‐measures analyses and post hoc comparisons were conducted in IBM SPSS Statistics version 27, and NBS permutation and Spearman correlation analyses were conducted in Python version 3.12.

## 3 Results

### 3.1 Study Participants and Baseline Characteristics

A total of 85 patients were included in the complete‐case analysis, including 42 in the active tACS group and 43 in the sham group (Figure 1). The two groups were comparable in demographics: age (73.48 ± 5.79 vs. 72.02 ± 7.18 years, *p =* 0.307), sex distribution (61.9% vs. 58.1% female, *p =* 0.894), years of education (6.79 ± 1.72 vs. 6.49 ± 1.59, *p =* 0.411), and body mass index (21.81 ± 3.59 vs. 23.10 ± 3.85 kg/m^2^, *p =* 0.115). There were also no significant between‐group differences in smoking, alcohol use, or tea consumption.

At baseline, cognitive performance and resting‐state FC did not differ significantly between groups. MMSE (21.88 ± 5.65 vs. 22.16 ± 5.28, *p* = 0.813) and total ADAS‐Cog scores (23.89 ± 10.68 vs. 24.13 ± 13.28, *p =* 0.926), as well as ADAS‐Cog subdomain scores for attention, language, memory, and praxis (all *p* > 0.05), were comparable. Likewise, the mean ROI–ROI resting‐state FC was similar between the active and sham groups (0.316 ± 0.158 vs 0.356 ± 0.177, *p =* 0.277) (Table 1).

**TABLE 1:** Baseline demographic, clinical, and functional connectivity characteristics of the active and sham groups.

Age	Active group (*n* = 42)	Sham group (*n* = 43)	t/χ^2^ value	*p* value
Age (y)	73.48 ± 5.79	72.02 ± 7.18	1.028	0.307
Gender (female)†	26 (61.9%)	25 (58.1%)	0.018	0.894
Education (y)	6.79 ± 1.72	6.49 ± 1.59	0.827	0.411
BMI (kg/m^2^)	21.81 ± 3.59	23.10 ± 3.85	−1.593	0.115
Smoke†			2.984	0.225
Never	13(31.0%)	21(48.8%)		
Occasionally	21(50.0%)	17(39.5%)		
Often	8(19.0%)	5(11.6%)		
Drink†			1.176	0.555
Never	13(31.0%)	18(41.9%)		
Occasionally	23(54.8%)	19(44.2%)		
Often	6(14.3%)	6(14.0%)		
Tea†			2.274	0.321
Never	13(31.0%)	14(32.6%)		
Occasionally	19(45.2%)	24(55.8%)		
Often	10(23.8%)	5(11.6%)		
ADAS‐Cog total	23.89 ± 10.68	24.13 ± 13.28	−0.093	0.926
ADAS‐Cog attention	0.90 ± 0.85	1.12 ± 1.40	−0.844	0.402
ADAS‐Cog language	4.60 ± 3.13	5.14 ± 4.39	−0.659	0.512
ADAS‐Cog memory	16.17 ± 6.65	15.83 ± 7.52	0.224	0.823
ADAS‐Cog praxis	2.21 ± 1.77	2.05 ± 1.27	0.500	0.618
MMSE total	21.88 ± 5.65	22.16 ± 5.28	−0.237	0.813
Functional connectivity	0.316±0.158	0.356±0.177	−1.095	0.277

Data are presented as mean ± SD or number (%).

Abbreviations: ADAS‐Cog, Alzheimer's Disease Assessment Scale–Cognitive Subscale; BMI, body mass index; FC, functional connectivity; MMSE, Mini‐Mental State Examination; ROI, region of interest; SD, standard deviation; zFC, Fisher z‐transformed functional connectivity.

### 3.2 Cognitive Outcomes

**FIGURE 2:**
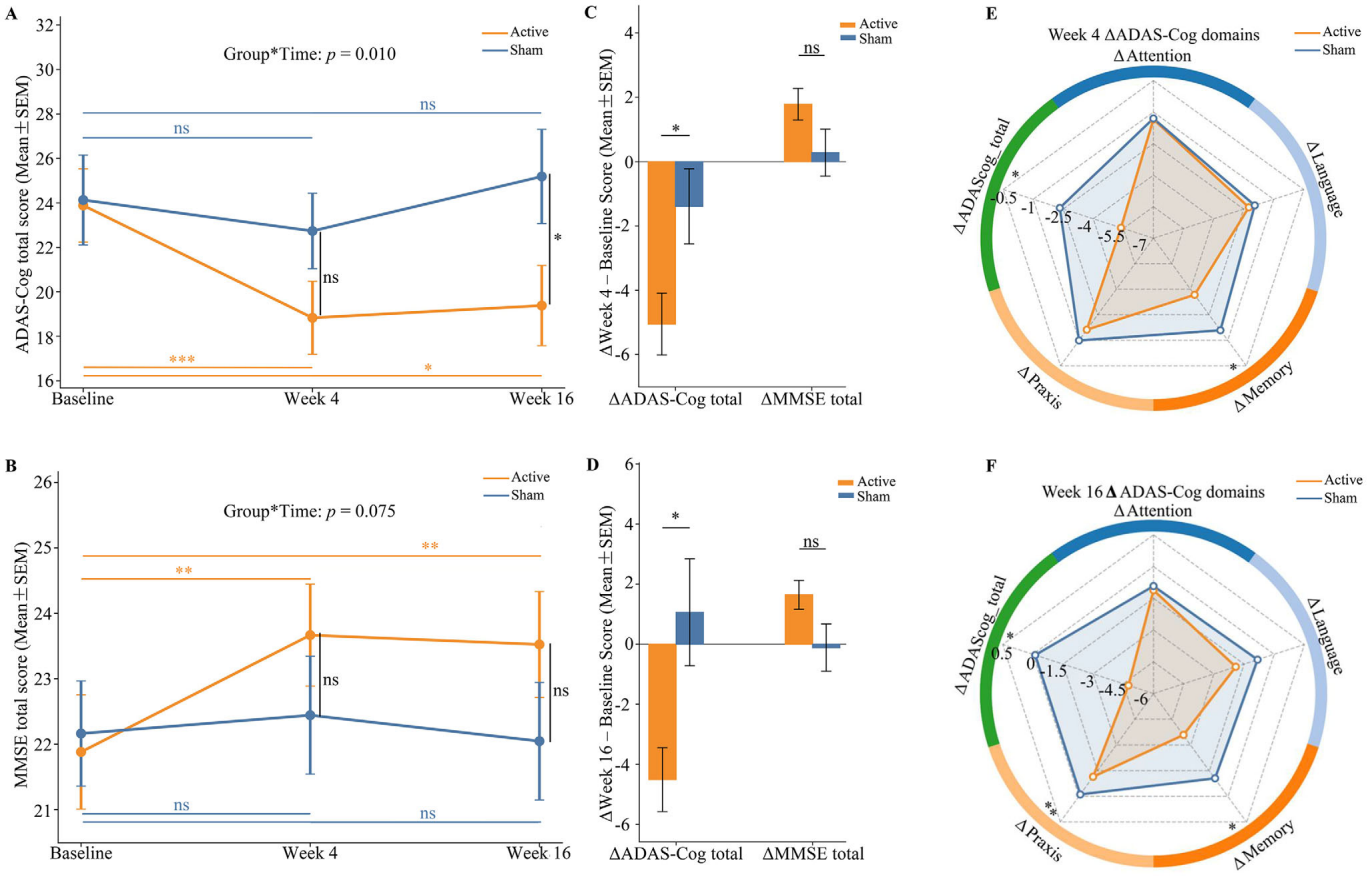
Longitudinal cognitive changes and domain‐level treatment effects. (A) Changes in the total ADAS‐Cog scores between the active treatment group and the sham tACS group at baseline, Week 4, and Week 16. Data are presented as mean ± SEM; orange and blue curves represent the active treatment group and the sham treatment group, respectively. The Group × Time interaction *p*‐value is shown in each panel. Colored horizontal lines indicate within‐group comparisons between baseline and corresponding follow‐up time points, while black vertical lines represent between‐group comparisons at Week 4 or Week 16. All post hoc pairwise‐comparison *p*‐values were Bonferroni‐adjusted. (B) Changes in total MMSE scores across three time points. The group × time interaction on MMSE scores was not statistically significant; within‐group MMSE comparisons are presented only for exploratory analysis purposes. (C) Between‐group changes in total ADAS‐Cog and MMSE scores compared to baseline at Week 4; (D) Between‐group changes in total ADAS‐Cog and MMSE scores compared to baseline at Week 16; (E) Radar plot illustrating trends in total ADAS‐Cog scores and their subscale scores at Week 4; (F) Radar plot illustrating trends in total ADAS‐Cog scores and their subscale scores at Week 16. P‐values in panels C–F were adjusted for false discovery rate (FDR). ns: no statistical significance; ^*^ *p* < 0.05, ^**^ *p* < 0.01, ^***^ *p* < 0.001. **Abbreviations**: ADAS‐Cog, Alzheimer's Disease Assessment Scale–Cognitive Subscale; MMSE, Mini‐Mental State Examination; SEM, standard error of the mean.

ADAS‐Cog total score showed a significant main effect of time (F_1.696,140.738_ = 6.788, *p* = 0.003) and a significant time × group interaction (F_1.696, 140.738_ = 5.215, *p* = 0.010, ηp^2^ = 0.059), while the main effect of group was not significant (F_1,83_ = 1.931, *p* = 0.168) (Table 2). In the active group, total ADAS‐Cog scores decreased from baseline (23.89 ± 10.68) to Week 4 (18.83 ± 10.63), and this improvement was maintained at Week 16 (19.38 ± 11.69; Table 2). The simple effect of Time was significant in the active group (*p* < 0.001; Table S2). Bonferroni‐adjusted pairwise comparisons showed mean reductions of 5.057 points from baseline to Week 4 (*p* < 0.001, Cohen's dz = 0.720) and 4.507 points from baseline to Week 16 (*p* = 0.010, Cohen's dz = 0.460; Figure 2A). Relative to baseline, the ADAS‐Cog total score improved by approximately 21.2% at Week 4 and 18.9% at Week 16. In the sham group, the score changed from 24.13 ± 13.28 at baseline to 22.74 ± 11.11 at Week 4 and 25.19 ± 13.90 at Week 16. The simple effect of Time was not significant (*p* = 0.058). Change‐score analyses showed greater reductions in the active group at Week 4 (t = −2.419, *p_FDR_* = 0.045) and Week 16 (t = −2.680, *p_FDR_* = 0.015; Figure 2C,D and Table S3). At Week 16, the ADAS‐Cog total score was lower in the active group than in the sham group (*p*_Bonferroni_ = 0.040).

**TABLE 2:** Longitudinal cognitive outcomes and mixed repeated‐measures analysis of variance results.

Outcome	Active Baseline	Active Week 4	Active Week 16	Sham Baseline	Sham Week 4	Sham Week 16	Group	Time	Group × Time	ηp^2^
ADAS‐Cog total	23.887±10.677	18.830±10.625	19.380±11.691	24.130±13.280	22.737±11.111	25.188±13.896	F(1,83) = 1.931, p = 0.168	F(1.696,140.738) = 6.788, p = 0.003	F(1.696,140.738) = 5.215, p = 0.010	0.059
MMSE	21.881±5.653	23.667±5.049	23.524±5.246	22.163±5.278	22.442±5.905	22.047±5.892	F(1,83) = 0.528, p = 0.469	F(1.281,106.343) = 3.825, p = 0.043	F(1.281,106.343) = 3.017, p = 0.075	0.035
Praxis^a^	2.214±1.774	1.667±1.004	2.048±1.724	2.047±1.272	2.269±1.544	3.116±1.942	F(1,83) = 3.201, p = 0.077	F(2,166) = 6.882, p = 0.001	F(2,166) = 6.494, p = 0.002	0.073
Memory	16.173±6.647	13.164±6.755	13.071±7.102	15.828±7.524	15.319±7.210	15.770±7.446	F(1,83) = 1.087, p = 0.300	F(1.689,140.170) = 8.065, p<0.001	F(1.689,140.170) = 5.670, p = 0.007	0.064
Attention	0.905±0.850	0.691±0.841	0.762±0.850	1.116±1.401	0.954±1.068	1.209±1.245	F(1,83) = 2.531, p = 0.115	F(1.642,136.322) = 1.716, p = 0.189	F(1.642,136.322) = 0.629, p = 0.504	0.008
Language	4.595±3.132	3.310±3.280	3.500±3.307	5.140±4.394	4.210±2.972	5.349±4.380	F(1,83) = 2.583, p = 0.112	F(1.650,136.932) = 5.404, p = 0.009	F(1.650,136.932) = 1.976, p = 0.151	0.023

^a^ The sphericity assumption was satisfied, and results from the sphericity‐assumed tests were reported.

Data are presented as mean ± SD.

Abbreviations: ADAS‐Cog, Alzheimer's Disease Assessment Scale–Cognitive Subscale; ANOVA, analysis of variance; MMSE, Mini‐Mental State Examination; SD, standard deviation; ηp^2^, partial eta squared.

Memory, F_1.689, 140.170_ = 5.670, *p* = 0.007, ηp^2^ = 0.064, and praxis, F_2, 166_ = 6.494, *p* = 0.002, ηp^2^ = 0.073, also showed significant Group × Time interactions. Reductions in memory score were greater in the active group at Week 4 (*p_FDR_* = 0.045) and Week 16 (*p_FDR_* = 0.015). The between‐group difference in praxis change was not significant at Week 4 (*p_FDR_* = 0.056) but was significant at Week 16 (*p_FDR_* = 0.009; Figure 2E,F). Group × Time interactions were not significant for MMSE total (F_1.281, 106.343_ = 3.017, *p* = 0.075, ηp^2^ = 0.035) (Figure 2B), language (F_1.650, 136.932_ = 1.976, *p* = 0.151, ηp^2^ = 0.023), or attention (F_1.642, 136.322_ = 0.629, *p* = 0.504, ηp^2^ = 0.008).

### 3.3 fNIRS Functional Connectivity

**FIGURE 3:**
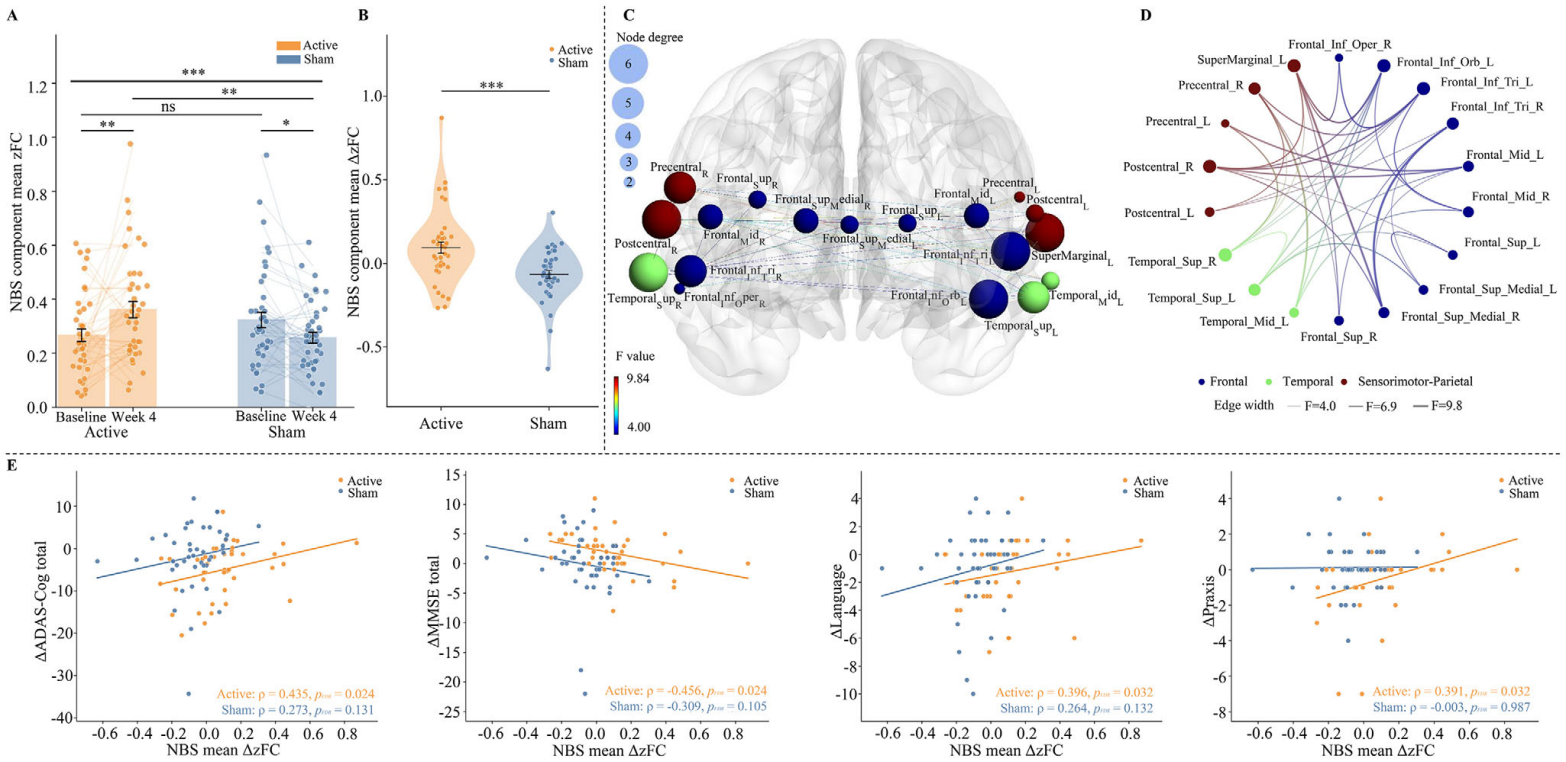
Functional connectivity network and its associations with cognitive score changes. (A) Pre–post distributions of mean zFC within the significant NBS component in the active and sham groups. Dots represent individual participants, connecting lines indicate paired observations, and bars and error bars represent the mean ± SEM. (B) Violin plots showing the distribution of mean connectivity changes within the significant NBS component. Dots represent individual participants, violin width indicates the estimated data density, and black horizontal lines and error bars represent the mean ± standard error of the mean (SEM). (C) Three‐dimensional brain visualization of the significant Group × Time interaction component identified by NBS. Node size represents node degree, and edge color represents the edge‐wise F statistic according to the color bar. (D) Circular network representation of the significant component across frontal, temporal, and sensorimotor–parietal regions. Edge width is proportional to the edge‐wise F statistic for the Group × Time interaction, and edge color corresponds to the anatomical module colors used in the network layout. (E) Scatter plots showing the associations between mean NBS‐derived (ΔzFC) and changes in ADAS‐Cog total, MMSE total, language, and praxis scores. Orange dots and regression lines represent the active group, whereas blue dots and regression lines represent the sham group. Spearman correlation coefficients (ρ) and FDR‐adjusted *p*‐values are shown in each panel. (^*^ *p* < 0.05, ^**^ *p* < 0.01, ^***^ *p* < 0.001). **Abbreviations**: ADAS‐Cog, Alzheimer's Disease Assessment Scale–Cognitive Subscale; FC, functional connectivity; MMSE, Mini‐Mental State Examination; NBS, network‐based statistic; SEM, standard error of the mean; zFC, Fisher z‐transformed functional connectivity.

NBS identified one significant component comprising 38 connections and 18 cortical nodes (component‐level *p_FWE_* = 0.010; Figure S1). Mean zFC within the component showed a significant Group × Time interaction (F_1, 83_ = 15.497, *p* < 0.001, ηp^2^ = 0.157) (Figure 3A), whereas the main effects of Group (F_1, 83_ = 0.613, *p* = 0.436), and Time (F_1, 83_ = 0.419, *p* = 0.519) were not significant (Table S4). Mean zFC increased from 0.267 ± 0.150 to 0.361 ± 0.197 in the active group (*p_Bonferroni_* = 0.002) and decreased from 0.323 ± 0.186 to 0.257 ± 0.133 in the sham group (*p_Bonferroni_* = 0.024). The groups did not differ at baseline (*p_Bonferroni_* = 0.127), whereas post‐intervention mean zFC was higher in the active group (*p_Bonferroni_* = 0.005, Cohen's d = 0.630). Change‐score analysis likewise showed a greater increase in mean network connectivity in the active group (t = 3.922, *p* < 0.001; Figure 3B).

The significant component involved the bilateral superior, middle, and inferior frontal gyri; bilateral superior temporal gyri; left middle temporal gyrus; bilateral precentral and postcentral gyri; and left supramarginal gyrus. The strongest edge‐level interaction statistics were observed for the right triangular inferior frontal gyrus–right medial superior frontal gyrus (F = 9.841, *p* = 0.002), right medial superior frontal gyrus–left supramarginal gyrus (F = 9.314, *p* = 0.003), right precentral gyrus–left middle temporal gyrus (F = 8.671, *p* = 0.004), and left middle frontal gyrus–left precentral gyrus (F = 7.856, *p* = 0.006; Figure 3C,D and Table S5). Additional exploratory analyses are provided in Table S6 and Figure S2.

### 3.4 Brain‐Behavior Correlations

In the active group, mean ΔzFC within the NBS‐defined component was significantly associated with changes in ADAS‐Cog total (ρ = 0.435, *p_FDR_* = 0.024), language (ρ = 0.396, *p_FDR_* = 0.032), praxis (ρ = 0.391, *p_FDR_* = 0.032), and MMSE total scores (ρ = −0.456, *p_FDR_* = 0.024). Associations with attention (ρ = 0.277, *p_FDR_* = 0.131) and memory (ρ = 0.162, *p_FDR_* = 0.365) were not significant. In the sham group, no association remained significant after FDR correction, including that between mean ΔzFC and ADAS‐Cog total change (ρ = 0.273, *p_FDR_* = 0.131; Figure 3E and Table S7).

### 3.5 Safety

Throughout the study period (4‐week intervention plus 3‐month follow‐up), no SAEs were reported in either group. The incidence of mild AEs was low; pruritus and fatigue were relatively more common in both groups but transient. Additionally, in the active group, 2 cases (4.76%) of mild headache and 5 cases of dizziness (11.90%) were reported; in the sham group, 3 cases of mild headache (6.98%) and 3 cases of dizziness (6.98%) were observed (Table S8). All these events resolved spontaneously within 1 h after the intervention, without the need for additional medical management.

## 4 Discussion

In this randomized, double‐blind, sham‐controlled trial, twice‐daily bilateral temporal 40‐Hz tACS administered for 4 consecutive weeks was associated with improved cognitive trajectories and altered FC within the FTPN in patients with AD. Cognitive benefits were primarily observed in global cognition, memory, and praxis as assessed by the ADAS‐Cog, and the improvement in global cognition remained relatively stable during follow‐up. Network analysis further showed that tACS‐related connectivity changes were not confined to the bilateral temporal targets but extended across a distributed network involving frontal, temporal, parietal, and sensorimotor cortices. Mean FC within this network increased after active stimulation but decreased after sham stimulation. In addition, changes in ADAS‐Cog total, language, praxis, and MMSE scores were significantly associated with changes in network connectivity. Collectively, these findings suggest that an accelerated, twice‐daily bilateral temporal 40‐Hz tACS protocol improved cognitive function in patients with AD and was accompanied by enhanced functional integration within the FTPN.

### 4.1 Cognitive Effects of Accelerated 40‐Hz tACS

The cognitive benefits observed in the present study are broadly consistent with those reported in previous studies of 40‐Hz tACS, particularly with respect to global cognitive performance measured by the ADAS‐Cog. Benussi et al. showed that a single session of precuneus‐targeted gamma‐tACS improved immediate, delayed, and associative memory in patients with AD or mild cognitive impairment and enhanced short‐latency afferent inhibition [23, 24]. Sprugnoli et al. applied repeated 40‐Hz tACS predominantly over the temporal regions. Although that study did not establish a definitive effect on global cognition, it demonstrated increased bilateral temporal perfusion, and the perfusion changes were associated with changes in episodic memory [7]. More recently, a DLPFC‐targeted trial also reported a more favorable trajectory of global cognitive change in the active‐stimulation group, accompanied by increased hippocampal–prefrontal connectivity and enhanced local low‐gamma connectivity [8].

Across cognitive domains, the treatment effects in the present study were mainly evident in the ADAS‐Cog total score, memory, and praxis, whereas language, attention, and MMSE did not show clear between‐group effects after correction for multiple comparisons. The improvement in memory is consistent with the established role of the temporal lobe in memory processing [25]. The more favorable praxis trajectory in the active group appeared to reflect attenuation of the deterioration observed in the sham group rather than a significant within‐group improvement. This relative preservation may be related to distributed modulation of frontoparietal and sensorimotor networks [26, 27]. The present study used a dose‐intensive protocol consisting of 2 mA stimulation twice daily for 4 weeks, resulting in a greater cumulative number of sessions and a longer overall study period than in several previous investigations. The persistence of ADAS‐Cog improvement through Week 16 suggests that repeated stimulation may have produced cumulative and sustained after‐effects.

### 4.2 Network‐Level Modulation of Functional Connectivity

Previous structural neuroimaging studies have shown that AD‐related brain atrophy and white matter damage are not confined to isolated regions but are distributed across interconnected frontal, temporal, and parietal networks and are associated with impairments in memory, language, and activities of daily living [12, 28, 29, 30]. The structural and functional integrity of the FTPN may therefore represent an important network‐level substrate of cognitive impairment in AD. Another major finding of the present study was that 40‐Hz tACS induced distributed FC changes extending beyond the local stimulation targets. The significant network component identified by NBS involved the bilateral superior, middle, and inferior frontal gyri, bilateral superior temporal gyri, left middle temporal gyrus, supramarginal gyrus, and precentral and postcentral gyri. These findings indicate that treatment‐related changes were not restricted to regions beneath the stimulation electrodes but involved broader coordination among frontal, temporal, parietal, and sensorimotor areas. The observed increase in connectivity may therefore reflect altered integration within distributed cortical systems supporting cognition rather than a purely local change in activity within a single brain region. This interpretation is also consistent with previous neuroimaging and electrophysiological findings [7, 8, 9].

### 4.3 Brain–Behavior Associations Following 40‐Hz tACS

Brain–behavior correlation analyses showed that, in the active‐stimulation group, mean ΔzFC within the NBS‐derived network was significantly associated with changes in the ADAS‐Cog total, language, praxis, and MMSE scores, whereas no significant correlations survived FDR correction in the sham group. These findings indicate statistical covariation between changes in network connectivity and changes in selected cognitive outcomes. From a functional perspective, frontal and parietal regions jointly contribute to cognitive control, attentional orienting, and goal‐directed behavior [31, 32], whereas temporal regions are closely involved in memory and language processing [33]. The precentral and postcentral gyri and the supramarginal gyrus also participate in sensorimotor integration, action selection, and the planning and execution of learned movements [34]. Therefore, the functional composition of the identified neural network in this study exhibits a certain degree of neuroanatomical consistency with changes in linguistic and practical abilities. Overall, these findings suggest that the cognitive benefits associated with 40‐Hz tACS may be accompanied by altered functional coordination across frontal, temporal, parietal, and sensorimotor regions. They also provide preliminary network‐level evidence for the potential neural mechanisms underlying the clinical effects of bilateral temporal 40‐Hz tACS.

### 4.4 Clinical Feasibility and Safety

From the perspective of clinical implementation, localization using T7/T8 of the International 10–20 system avoids the need for magnetic resonance imaging (MRI)‐guided neuronavigation and facilitates standardized, repeatable delivery. No serious adverse events occurred, and reported headache, dizziness, pruritus, and fatigue were generally mild and transient, consistent with previous safety data for low‐intensity transcranial electrical stimulation and tACS studies in AD [7, 8, 9, 19]. These observations support the preliminary feasibility and tolerability of the present protocol.

Although the present study provides preliminary evidence for the efficacy of accelerated 40 Hz tACS in AD, several limitations should be acknowledged. First, this was a single‐center randomized controlled trial. Despite an a priori sample size calculation, the overall sample size remains limited, and its generalizability needs to be further validated by multicenter, large‐sample studies. Second, AD diagnosis was based primarily on clinical criteria. Future studies should incorporate structural MRI and molecular biomarkers to improve diagnostic precision, while also including standardized clinical staging to further evaluate potential differences in treatment efficacy across disease stages. Third, the current study only evaluated hemodynamic FC on the cortical surface using fNIRS, which cannot directly measure the activity of deep brain structures nor simultaneously record EEG or transcranial magnetic stimulation‐EEG. Thus, the electrophysiological characteristics of gamma‐band entrainment remain inferential, and future studies may adopt multimodal imaging to obtain more comprehensive mechanistic evidence. Finally, correlations based on change scores cannot establish causality or mediation. Larger prospective studies should compare stimulation intensity, treatment frequency, and intersession intervals and integrate individualized structural imaging and electric‐field modeling to optimize treatment.

## 5 Conclusions

Bilateral temporal 40‐Hz tACS delivered at 2 mA twice daily for 4 weeks was associated with sustained improvement in ADAS‐Cog performance and with distributed cortical functional‐connectivity changes in patients with AD. These findings provide preliminary evidence that accelerated 40‐Hz tACS can modulate AD‐related cortical networks; however, frequency specificity, durability, and clinical scalability require confirmation in larger, multicenter, biomarker‐defined, multimodal studies.

## Author Contributions

**Rong Guo**: software, methodology, data curation, validation, formal analysis, visualization, writing – original draft, writing – review and editing. **Xingxing Li**: conceptualization, methodology, investigation, validation, data curation, supervision, writing – review and editing. **Chenjun Zou**: data curation. **Chao Zheng**: investigation. **Xiaohong Wu**: investigation. **Xuechao Lu**: data curation. **Jiaying Yan**: investigation. **Junfang Zhang**: supervision, project administration. **Xiaoyan Luo**: investigation. **Shiwei Ye**: conceptualization, data curation, methodology, supervision, investigation. **Dongsheng Zhou**: conceptualization, methodology, software, data curation, validation, formal analysis, supervision, funding acquisition, project administration, resources, writing – review and editing.

## Funding

This work was supported by the Ningbo Public Welfare Science and Technology Plan Project (Grant No. 2023S035), the Ningbo Medical and Health Brand Discipline (Grant No. PPXK20182024‐0708), the Ningbo Clinical Medical Research Centre for Mental Health (Grant No. 2022L002), the Ningbo Top Medical and Health Research Program (Grant No. 2022030410), and the Lishui Municipal Public Welfare Technology Application Research Program (Grant No. 2024GYX41).

## Conflicts of Interest

The authors declare no conflicts of interest.

## Supporting information




**Supporting File**: advs77851‐sup‐0001‐SuppMat.docx.

## Data Availability

The data that support the findings of this study are available on request from the corresponding author. The data are not publicly available due to privacy or ethical restrictions.
