# Supplementary material for: Temporal‐Targeted Accelerated 40 Hz tACS Enhances Cognitive Function and Network Integration in Alzheimer's Disease: A Randomized Clinical Trial
Source: Adv Sci (Weinh). 2026 Sep 27:e77851. Online ahead of print. doi: 10.1002/advs.77851 (PMC13616376; doi:10.1002/advs.77851)
Supplement: Supplementary file 1 [file ADVS-9999-e77851-s001.docx]

Supplementary Materials

[Supplementary Materials	1](#_Toc8435)

[Supplementary methods	2](#_Toc1148)

[1. Intervention Protocol	2](#_Toc19107)

[2. ICD-10 Diagnostic Criteria	2](#_Toc5080)

[3. Calculation of percentage improvement in ADAS-Cog total score：	3](#_Toc23410)

[4.ROI-level node localization for network visualization.	3](#_Toc30826)

[5. Adverse Event Monitoring	3](#_Toc28127)

[Supplementary tables	5](#_Toc29015)

[Supplementary Table 1. Anatomical ROI Assignments and MNI Coordinates of the fNIRS Measurement Channels	5](#_Toc8344)

[Supplementary Table 2. Simple-effects analyses and Bonferroni-adjusted pairwise comparisons for cognitive outcomes with significant Group × Time interaction.	7](#_Toc30282)

[Supplementary Table 3. Between-group comparisons of cognitive change scores from baseline to Week 4 and Week 16	10](#_Toc22920)

[Supplementary Table 4. Mean functional connectivity of the significant NBS component: mixed repeated-measures ANOVA and Bonferroni-adjusted simple-effects analyses.	11](#_Toc5826)

[Supplementary Table 5. Edge-wise Group × Time interaction statistics for all ROI-to-ROI connections.	12](#_Toc11881)

[Supplementary Table 6. Exploratory within-group pre–post changes in individual connections of the significant NBS component.	28](#_Toc11489)

[Supplementary Table 7. Associations between NBS-derived mean ΔzFC and cognitive score changes.	32](#_Toc1760)

[Supplementary Table 8. Adverse events and tolerability during 40-Hz tACS	34](#_Toc18333)

[Supplementary figures	35](#_Toc27750)

[Figure S1. Functional connectivity changes and the significant NBS component.	35](#_Toc3966)

[Figure S2. Exploratory within-group functional connectivity changes within the significant NBS component	36](#_Toc24203)

[Reference	37](#_Toc16979)

# Supplementary methods

## 1. Intervention Protocol

Two saline-soaked sponge electrodes (4 × 4 cm²) were positioned over the bilateral temporal regions at T7 and T8 according to the International 10–20 EEG system, aiming to achieve standardized scalp localization of the bilateral temporal areas. A 40-Hz sinusoidal alternating current was delivered using a transcranial stimulator (Kerfun Medical Co., Ltd., Xi’an, China) at an intensity of 2 mA (peak amplitude, zero-to-peak) for 20 min per session. During transcranial alternating current stimulation (tACS), the direction of the current reverses every half cycle [1].

Participants in the sham group received stimulation using the same device but with a sham protocol. At the beginning of each session, the current was ramped up to 2 mA over 3 s, maintained at this intensity for 30 s, and then gradually ramped down to 0 mA. This ramp-up/ramp-down procedure was intended to mimic the initial somatic sensations produced by active stimulation, such as tingling and itching, while minimizing sustained neuromodulatory effects [1-2]. This procedure was designed to prevent participants from distinguishing active from sham stimulation based on the appearance of the device or their subjective sensations [3]. The sham group followed the same treatment schedule as the active group: twice daily, 5 days per week, with an interval of at least 4 h between sessions, for a total of 40 sessions over 4 consecutive weeks.

Participants received two 20-min stimulation sessions per day, separated by an interval of at least 4 hours. This schedule was informed by previous clinical studies employing twice-daily transcranial electrical stimulation protocols [4] and by evidence that the effects of repeated stimulation are time dependent. Excessively short intersession intervals may result in nonlinear interactions, attenuation of stimulation effects, or homeostatic-like and metaplastic responses between consecutive sessions [5-6]. Taking into account patients’ daily treatment schedules, rest requirements, and the feasibility of completing a 4-week intervention, a minimum interval of 4 hours was selected to maintain a dose-intensive twice-daily protocol while minimizing direct interference between consecutive stimulation sessions.

## 2. ICD-10 Diagnostic Criteria

According to the ICD-10 diagnostic criteria for dementia in Alzheimer’s disease (F00), the following conditions should be met: the presence of a dementia syndrome; an insidious onset with slow and progressive cognitive decline; no clinical evidence that the cognitive impairment is better explained by another cerebral or systemic disorder; and no sudden apoplectic onset or prominent focal neurological signs during the early stage of the disease. Accordingly, the diagnostic assessment in the present study was based on the patient’s medical history, the course of cognitive decline, cognitive and functional scale assessments, and clinical physical examinations, together with the available clinical information to exclude other potential causes of cognitive impairment.

## 3. Calculation of percentage improvement in ADAS-Cog total score：

Percentage improvement from Baseline to Week 4：

$$\text{Percentage}\text{ }\text{of}\text{ }\text{score}\text{ }\text{improvement}\text{ =}\frac{\text{Baseline}_{\text{Mean}}\text{-}\text{Week}\text{4}_{\text{Mean}}}{\text{Baseline}_{\text{Mean}}}\text{ ×100\%}$$

Percentage improvement from Baseline to Week 16：

$$\text{Percentage}\text{ }\text{of}\text{ }\text{score}\text{ }\text{improvement}\text{ =}\frac{\text{Baseline}_{\text{Mean}}\text{-}\text{Week}\text{16}_{\text{Mean}}}{\text{Baseline}_{\text{Mean}}}\text{ ×100\%}$$

## 4.ROI-level node localization for network visualization.

For ROI-level visualization of the NBS-derived network, a single representative MNI coordinate was required for each anatomical ROI. When an ROI was represented by a single fNIRS measurement channel, the MNI coordinate provided for that channel was used directly. When multiple measurement channels were assigned to the same ROI, the x-, y-, and z-coordinates of all corresponding channels were averaged separately to derive a representative ROI-level MNI coordinate. Thus, the resulting node location represents the spatial mean of the actual fNIRS sampling positions assigned to that ROI rather than the geometric centroid of the entire atlas-defined anatomical region [7].

## 5. Adverse Event Monitoring

During the intervention period, safety was monitored using a standardized adverse-event checklist completed after each transcranial alternating current stimulation (tACS) session. Participants and their caregivers were instructed to promptly report any new or worsening symptoms. All adverse events were documented and graded according to the Common Terminology Criteria for Adverse Events, version 5.0 (CTCAE v5.0): Grade 1, mild (asymptomatic or mild symptoms; clinical or diagnostic observations only; intervention not indicated); Grade 2, moderate; and Grade 3, severe [8]. The relationship between each adverse event and the intervention was also assessed, and all adverse events were followed until complete resolution. In the event of a serious adverse event, immediate clinical management was initiated, tACS was adjusted or discontinued as appropriate, or the participant was withdrawn from the study and continued to receive medical follow-up.

# Supplementary tables

## Supplementary Table 1. Anatomical ROI Assignments and MNI Coordinates of the fNIRS Measurement Channels

| ROI | channel | MNI_x | MNI_y | MNI_z |
| --- | --- | --- | --- | --- |
| Superior Temporal Gyrus (Right) | 01 | 71 | -11.3333 | -10.3333 |
| Superior Temporal Gyrus (Right) | 02 | 73 | -24.3333 | 1.3333 |
| Inferior Frontal Gyrus, Opercular Part (Right) | 03 | 61 | 12.3333 | -10.3333 |
| Inferior Frontal Gyrus, Triangular Part (Right) | 04 | 55 | 40.6667 | -9.3333 |
| Inferior Frontal Gyrus, Triangular Part (Right) | 05 | 59 | 26.3333 | 1.3333 |
| Middle Frontal Gyrus (Right) | 06 | 41.6667 | 61.3333 | -8.3333 |
| Superior Frontal Gyrus, Medial Part (Right) | 07 | 15.3333 | 72 | -8.3333 |
| Superior Frontal Gyrus (Right) | 08 | 31.6667 | 68 | 1.3333 |
| Superior Frontal Gyrus (Left) | 09 | -17 | 69.6667 | -10.3333 |
| Middle Frontal Gyrus (Left) | 10 | -42.6667 | 56.3333 | -9.6667 |
| Superior Frontal Gyrus (Left) | 11 | -29 | 67 | -1 |
| Inferior Frontal Gyrus, Orbital Part (Left) | 12 | -49 | 23 | -14 |
| Superior Temporal Gyrus (Left) | 13 | -65 | -0.3333 | -13.3333 |
| Inferior Frontal Gyrus, Triangular Part (Left) | 14 | -56.6667 | 12.3333 | -3.6667 |
| Middle Temporal Gyrus (Left) | 15 | -71 | -22.3333 | -12.6667 |
| Middle Temporal Gyrus (Left) | 16 | -71 | -32.6667 | -2.3333 |
| Postcentral Gyrus (Right) | 17 | 65 | 1.6667 | 2 |
| Postcentral Gyrus (Right) | 18 | 70 | -8.6667 | 14.3333 |
| Precentral Gyrus (Right) | 19 | 63.6667 | 13.3333 | 11.3333 |
| Postcentral Gyrus (Right) | 20 | 67 | 1.3333 | 26.6667 |
| Middle Frontal Gyrus (Right) | 21 | 50.6667 | 50.3333 | 2.3333 |
| Middle Frontal Gyrus (Right) | 22 | 56.3333 | 38.3333 | 13.3333 |
| Middle Frontal Gyrus (Right) | 23 | 42 | 59.6667 | 13 |
| Middle Frontal Gyrus (Right) | 24 | 50 | 44.6667 | 23.6667 |
| Superior Frontal Gyrus, Medial Part (Left) | 25 | -0.6667 | 69 | -0.6667 |
| Superior Frontal Gyrus, Medial Part (Right) | 26 | 16.3333 | 73 | 11.3333 |
| Superior Frontal Gyrus (Left) | 27 | -16 | 72.3333 | 11.3333 |
| Superior Frontal Gyrus, Medial Part (Left) | 28 | 1.6667 | 64.3333 | 25.6667 |
| Middle Frontal Gyrus (Left) | 29 | -51.3333 | 45.6667 | -1.6667 |
| Middle Frontal Gyrus (Left) | 30 | -40.3333 | 58.6667 | 10.6667 |
| Inferior Frontal Gyrus, Triangular Part (Left) | 31 | -57 | 30.6667 | 9.6667 |
| Middle Frontal Gyrus (Left) | 32 | -48.3333 | 42.6667 | 22.3333 |
| Postcentral Gyrus (Left) | 33 | -68 | -12 | -1 |
| Postcentral Gyrus (Left) | 34 | -64 | 1.6667 | 8.6667 |
| Supramarginal Gyrus (Left) | 35 | -69 | -21.3333 | 9.6667 |
| Postcentral Gyrus (Left) | 36 | -68 | -10.6667 | 23.6667 |
| Middle Frontal Gyrus (Right) | 37 | 59 | 24.6667 | 25.6667 |
| Precentral Gyrus (Right) | 38 | 58 | 10.6667 | 40 |
| Middle Frontal Gyrus (Right) | 39 | 50.3333 | 31.3333 | 37.3333 |
| Superior Frontal Gyrus (Right) | 40 | 30.6667 | 60.6667 | 25.6667 |
| Superior Frontal Gyrus (Right) | 41 | 37.3333 | 46.3333 | 37.3333 |
| Superior Frontal Gyrus, Medial Part (Right) | 42 | 16.3333 | 58.3333 | 38.6667 |
| Superior Frontal Gyrus (Left) | 43 | -26.3333 | 61.3333 | 25.3333 |
| Superior Frontal Gyrus (Left) | 44 | -12.3333 | 58.3333 | 39.6667 |
| Middle Frontal Gyrus (Left) | 45 | -34.6667 | 45.6667 | 37.6667 |
| Precentral Gyrus (Left) | 46 | -60 | 16.3333 | 22.3333 |
| Middle Frontal Gyrus (Left) | 47 | -51 | 27.6667 | 34.6667 |
| Postcentral Gyrus (Left) | 48 | -62 | 1.6667 | 34.6667 |

Note: Anatomical ROI assignments and MNI coordinates were obtained from the spatial localization information provided by the fNIRS system and were used to determine the representative ROI-level coordinates for network visualization.

## Supplementary Table 2. Simple-effects analyses and Bonferroni-adjusted pairwise comparisons for cognitive outcomes with significant Group × Time interaction.

| Outcome |  | Post-hoc comparison | Mean | SE | p | 95%CI | Post-hoc F | Post-hoc P | ηp² | Cohen's d_z_/d |
| --- | --- | --- | --- | --- | --- | --- | --- | --- | --- | --- |
|  |  |  |  |  |  |  | 10.827 | <0.0001 | 0.209 |  |
| ADAS-cog total | active | Baseline vs Week 4 | 5.057 | 1.080 | <0.001 | [2.417,7.696] |  |  |  | 0.720 |
|  |  | Baseline vs Week 16 | 4.507 | 1.485 | 0.010 | [0.877,8.136] |  |  |  | 0.460 |
|  |  | Week 4 vs  Week 16 | -0.550 | 1.133 | 1.000 | [-3.319,2.219] |  |  |  | -0.080 |
|  |  |  |  |  |  |  | 2.956 | 0.058 | 0.067 |  |
|  | sham | Baseline vs Week 4 | 1.393 | 1.067 | 0.587 | [-1.216,4.001] |  |  |  | 0.200 |
|  |  | Baseline vs Week 16 | -1.058 | 1.468 | 1.000 | [-4.645,2.529] |  |  |  | -0.110 |
|  |  | Week 4 vs  Week 16 | -2.451 | 1.120 | 0.094 | [-5.187,0.285] |  |  |  | -0.340 |
|  | Baseline | active vs sham | -0.243 | 2.617 | 0.926 | [-5.449,4.963] | 0.009 | 0.926 | 0.000 | -0.020 |
|  | Week 4 | active vs sham | -3.907 | 2.359 | 0.101 | [-8.599,0.785] | 2.743 | 0.101 | 0.032 | -0.350 |
|  | Week 16 | active vs sham | -5.808 | 2.789 | 0.040 | [-11.355,-0.262] | 4.338 | 0.040 | 0.050 | -0.440 |
|  |  |  |  |  |  |  | 3.065 | 0.052 | 0.070 |  |
| Praxis | active | Baseline vs Week 4 | 0.548 | 0.246 | 0.087 | [-0.054,1.15] |  |  |  | 0.340 |
|  |  | Baseline vs Week 16 | 0.167 | 0.272 | 1.000 | [-0.499,0.832] |  |  |  | 0.090 |
|  |  | Week 4 vs  Week 16 | -0.381 | 0.216 | 0.246 | [-0.909,0.148] |  |  |  | -0.270 |
|  |  |  |  |  |  |  | 10.457 | <0.001 | 0.203 |  |
|  | sham | Baseline vs Week 4 | -0.209 | 0.243 | 1.000 | [-0.804,0.386] |  |  |  | -0.130 |
|  |  | Baseline vs Week 16 | -1.070 | 0.269 | <0.001 | [-1.728,-0.412] |  |  |  | -0.610 |
|  |  | Week 4 vs  Week 16 | -0.860 | 0.214 | <0.001 | [-1.383,-0.338] |  |  |  | -0.620 |
|  | Baseline | active vs sham | 0.168 | 0.334 | 0.617 | [-0.497,0.833] | 0.252 | 0.617 | 0.003 | 0.110 |
|  | Week 4 | active vs sham | -0.589 | 0.283 | 0.041 | [-1.153,-0.026] | 4.325 | 0.041 | 0.050 | -0.440 |
|  | Week 16 | active vs sham | -1.069 | 0.399 | 0.009 | [-1.862,-0.276] | 7.185 | 0.009 | 0.080 | -0.570 |
|  |  |  |  |  |  |  | 9.562 | <0.001 | 0.189 |  |
| Memory | active | Baseline vs Week 4 | 3.009 | 0.704 | <0.001 | [1.289,4.729] |  |  |  | 0.650 |
|  |  | Baseline vs Week 16 | 3.102 | 0.794 | <0.001 | [1.161,5.043] |  |  |  | 0.590 |
|  |  | Week 4 vs  Week 16 | 0.093 | 0.533 | 1.000 | [-1.21,1.396] |  |  |  | 0.030 |
|  |  |  |  |  |  |  | 0.525 | 0.594 | 0.013 |  |
|  | sham | Baseline vs Week 4 | 0.509 | 0.696 | 1.000 | [-1.191,2.209] |  |  |  | 0.110 |
|  |  | Baseline vs Week 16 | 0.058 | 0.785 | 1.000 | [-1.86,1.976] |  |  |  | 0.010 |
|  |  | Week 4 vs  Week 16 | -0.451 | 0.527 | 1.000 | [-1.738,0.836] |  |  |  | -0.130 |
|  | Baseline | active vs sham | 0.345 | 1.541 | 0.824 | [-2.721,3.41] | 0.050 | 0.824 | 0.001 | 0.050 |
|  | Week 4 | active vs sham | -2.155 | 1.516 | 0.159 | [-5.171,0.86] | 2.020 | 0.159 | 0.024 | -0.280 |
|  | Week 16 | active vs sham | -2.699 | 1.579 | 0.091 | [-5.84,0.441] | 2.922 | 0.091 | 0.034 | -0.330 |

The table presents simple-effects analyses and pairwise comparisons for ADAS-Cog total, memory, and praxis scores. Mean difference was calculated as the first listed time point or group minus the second. Pairwise p values were adjusted using the Bonferroni method. Cohen’s dz is reported for within-group repeated comparisons, whereas Cohen’s d is reported for between-group comparisons.

Abbreviations: ADAS-Cog, Alzheimer’s Disease Assessment Scale–Cognitive Subscale; CI, confidence interval; d, Cohen’s effect size for independent groups; dz, Cohen’s effect size for paired observations; SE, standard error; ηp², partial eta squared.

## Supplementary Table 3. Between-group comparisons of cognitive change scores from baseline to Week 4 and Week 16

| **Time** | **Scale** | **Active delta mean±SD** | **Sham delta mean±SD** | **t** | **p_raw** | **p_FDR** |
| --- | --- | --- | --- | --- | --- | --- |
| **Week4-baseline** | Attention | -0.214±0.717 | -0.163±1.153 | -0.248 | 0.805 | 0.805 |
|  | Language | -1.286±2.298 | -0.93±2.915 | -0.625 | 0.534 | 0.667 |
|  | Memory | -3.009±4.103 | -0.509±4.969 | -2.532 | 0.013 | 0.045 |
|  | Praxis | -0.548±1.953 | 0.209±1.146 | -2.173 | 0.033 | 0.056 |
|  | ADAScog_total | -5.057±6.221 | -1.393±7.684 | -2.419 | 0.018 | 0.045 |
|  | MMSE_total | 1.786±3.205 | 0.279±4.788 | 1.709 | 0.092 | 0.092 |
| **Week16-baseline** | Attention | -0.143±0.814 | 0.093±1.525 | -0.893 | 0.375 | 0.375 |
|  | Language | -1.095±2.703 | 0.209±4.58 | -1.603 | 0.113 | 0.142 |
|  | Memory | -3.102±4.33 | -0.058±5.838 | -2.735 | 0.008 | 0.015 |
|  | Praxis | -0.167±1.82 | 1.07±1.71 | -3.226 | 0.002 | 0.009 |
|  | ADAScog_total | -4.507±6.904 | 1.058±11.688 | -2.680 | 0.009 | 0.015 |
|  | MMSE_total | 1.643±3.091 | -0.116±5.183 | 1.906 | 0.061 | 0.061 |

Data are presented as mean ± SD.

Abbreviations: ADAS-Cog, Alzheimer’s Disease Assessment Scale–Cognitive Subscale; FDR, false discovery rate; MMSE, Mini-Mental State Examination; SD, standard deviation

## Supplementary Table 4. Mean functional connectivity of the significant NBS component: mixed repeated-measures ANOVA and Bonferroni-adjusted simple-effects analyses.

| Active Pre | Active Post | Sham Pre | Sham Post | Group F, *p* value | Time F, *p* value | Group×Time F, *p* value | ηp² |  | Post-hoc comparison | Mean | SE | p | 95%CI | Post-hoc statistic (p) | Cohen's d_z_/d |
| --- | --- | --- | --- | --- | --- | --- | --- | --- | --- | --- | --- | --- | --- | --- | --- |
| 0.267±0.150 | 0.361±0.197 | 0.323±0.186 | 0.257±0.133 | F(1,83)=0.613, p=0.436 | F(1,83)=0.419, p=0.519 | F(1,83)=15.497, p<0.001 | 0.157 | active | Baseline vs Week 4 | -0.094 | 0.029 | 0.002 | [-0.152,  -0.037] | 10.596(0.002) | 0.502 |
|  |  |  |  |  |  |  |  | sham | Baseline vs Week 4 | 0.066 | 0.029 | 0.024 | [0.009,  0.123] | 5.320(0.024) | 0.352 |
|  |  |  |  |  |  |  |  | Baseline | Active vs sham | -0.056 | 0.037 | 0.127 | [-0.129,  0.016] | 2.381(0.127) | 0.340 |
|  |  |  |  |  |  |  |  | Week 4 | Active vs sham | 0.104 | 0.036 | 0.005 | [0.032,  0.176] | 8.154(0.005) | 0.630 |

Mean zFC values are presented as mean ± SD. Simple-effects p values were adjusted using the Bonferroni method. Cohen’s dz is reported for within-group comparisons, and Cohen’s d is reported for between-group comparisons.

Abbreviations: ANOVA, analysis of variance; CI, confidence interval; d, Cohen’s effect size for independent groups; dz, Cohen’s effect size for paired observations; FC, functional connectivity; FWE, family-wise error; NBS, network-based statistic; SD, standard deviation; SE, standard error; zFC, Fisher z-transformed functional connectivity; ηp², partial eta squared.

## Supplementary Table 5. Edge-wise Group × Time interaction statistics for all ROI-to-ROI connections.

| Connection | Active_Pre_mean_zFC | Active_Post_mean_zFC | Sham_Pre_mean_zFC | Sham_Post_mean_zFC | F_Group_x_Time | p_interaction_raw |
| --- | --- | --- | --- | --- | --- | --- |
| Inferior Frontal Gyrus, Triangular Part (Right) – Superior Frontal Gyrus, Medial Part (Right) | 0.225 | 0.401 | 0.285 | 0.230 | 9.841 | 0.002 |
| Superior Frontal Gyrus, Medial Part (Right) – Supramarginal Gyrus (Left) | 0.491 | 0.666 | 0.642 | 0.540 | 9.314 | 0.003 |
| Precentral Gyrus (Right) – Middle Temporal Gyrus (Left) | 0.104 | 0.214 | 0.194 | 0.098 | 8.671 | 0.004 |
| Middle Frontal Gyrus (Left) – Precentral Gyrus (Left) | 0.314 | 0.458 | 0.408 | 0.377 | 7.856 | 0.006 |
| Postcentral Gyrus (Right) – Inferior Frontal Gyrus, Triangular Part (Left) | 0.148 | 0.222 | 0.221 | 0.129 | 7.643 | 0.007 |
| Postcentral Gyrus (Right) – Middle Frontal Gyrus (Left) | 0.283 | 0.357 | 0.393 | 0.278 | 7.584 | 0.007 |
| Inferior Frontal Gyrus, Orbital Part (Left) – Supramarginal Gyrus (Left) | 0.384 | 0.480 | 0.453 | 0.383 | 7.173 | 0.009 |
| Inferior Frontal Gyrus, Opercular Part (Right) – Inferior Frontal Gyrus, Triangular Part (Left) | 0.212 | 0.356 | 0.279 | 0.225 | 6.951 | 0.010 |
| Precentral Gyrus (Right) – Inferior Frontal Gyrus, Triangular Part (Left) | 0.242 | 0.322 | 0.281 | 0.176 | 6.864 | 0.010 |
| Superior Temporal Gyrus (Right) – Precentral Gyrus (Left) | 0.459 | 0.587 | 0.500 | 0.454 | 6.664 | 0.012 |
| Superior Temporal Gyrus (Left) – Inferior Frontal Gyrus, Orbital Part (Left) | 0.257 | 0.367 | 0.325 | 0.297 | 6.634 | 0.012 |
| Postcentral Gyrus (Right) – Supramarginal Gyrus (Left) | 0.258 | 0.340 | 0.316 | 0.245 | 6.521 | 0.012 |
| Middle Temporal Gyrus (Left) – Inferior Frontal Gyrus, Orbital Part (Left) | 0.241 | 0.312 | 0.319 | 0.239 | 6.328 | 0.014 |
| Postcentral Gyrus (Right) – Inferior Frontal Gyrus, Orbital Part (Left) | 0.146 | 0.235 | 0.238 | 0.149 | 6.299 | 0.014 |
| Superior Frontal Gyrus, Medial Part (Right) – Postcentral Gyrus (Right) | 0.160 | 0.254 | 0.215 | 0.143 | 6.289 | 0.014 |
| Superior Temporal Gyrus (Left) – Superior Frontal Gyrus, Medial Part (Left) | 0.326 | 0.410 | 0.396 | 0.300 | 6.280 | 0.014 |
| Middle Frontal Gyrus (Right) – Inferior Frontal Gyrus, Triangular Part (Left) | 0.162 | 0.236 | 0.233 | 0.169 | 6.072 | 0.016 |
| Middle Frontal Gyrus (Right) – Middle Temporal Gyrus (Left) | 0.215 | 0.286 | 0.283 | 0.202 | 5.842 | 0.018 |
| Inferior Frontal Gyrus, Opercular Part (Right) – Supramarginal Gyrus (Left) | 0.135 | 0.196 | 0.200 | 0.110 | 5.774 | 0.018 |
| Superior Frontal Gyrus (Right) – Supramarginal Gyrus (Left) | 0.393 | 0.549 | 0.330 | 0.286 | 5.683 | 0.019 |
| Superior Temporal Gyrus (Left) – Middle Frontal Gyrus (Left) | 0.256 | 0.310 | 0.315 | 0.255 | 5.529 | 0.021 |
| Middle Frontal Gyrus (Right) – Inferior Frontal Gyrus, Orbital Part (Left) | 0.157 | 0.261 | 0.228 | 0.204 | 5.390 | 0.023 |
| Precentral Gyrus (Right) – Postcentral Gyrus (Left) | 0.239 | 0.342 | 0.300 | 0.281 | 5.359 | 0.023 |
| Superior Frontal Gyrus (Left) – Superior Frontal Gyrus, Medial Part (Left) | 0.173 | 0.306 | 0.232 | 0.176 | 5.109 | 0.026 |
| Inferior Frontal Gyrus, Triangular Part (Right) – Superior Frontal Gyrus (Right) | 0.234 | 0.331 | 0.237 | 0.181 | 4.971 | 0.028 |
| Superior Temporal Gyrus (Right) – Inferior Frontal Gyrus, Orbital Part (Left) | 0.179 | 0.272 | 0.231 | 0.189 | 4.772 | 0.032 |
| Superior Temporal Gyrus (Right) – Superior Temporal Gyrus (Left) | 0.174 | 0.244 | 0.227 | 0.170 | 4.727 | 0.033 |
| Superior Temporal Gyrus (Left) – Superior Frontal Gyrus (Left) | 0.232 | 0.290 | 0.291 | 0.243 | 4.587 | 0.035 |
| Superior Temporal Gyrus (Right) – Inferior Frontal Gyrus, Triangular Part (Left) | 0.194 | 0.263 | 0.226 | 0.166 | 4.563 | 0.036 |
| Inferior Frontal Gyrus, Triangular Part (Right) – Postcentral Gyrus (Left) | 0.289 | 0.335 | 0.349 | 0.282 | 4.435 | 0.038 |
| Superior Frontal Gyrus (Right) – Precentral Gyrus (Right) | 0.334 | 0.402 | 0.357 | 0.289 | 4.397 | 0.039 |
| Inferior Frontal Gyrus, Triangular Part (Left) – Postcentral Gyrus (Left) | 0.336 | 0.409 | 0.400 | 0.297 | 4.251 | 0.042 |
| Superior Temporal Gyrus (Right) – Supramarginal Gyrus (Left) | 0.438 | 0.540 | 0.467 | 0.407 | 4.216 | 0.043 |
| Postcentral Gyrus (Right) – Superior Frontal Gyrus (Left) | 0.466 | 0.622 | 0.406 | 0.393 | 4.157 | 0.045 |
| Superior Frontal Gyrus, Medial Part (Right) – Middle Frontal Gyrus (Left) | 0.370 | 0.481 | 0.427 | 0.381 | 4.133 | 0.045 |
| Superior Temporal Gyrus (Right) – Precentral Gyrus (Right) | 0.311 | 0.356 | 0.395 | 0.315 | 4.096 | 0.046 |
| Inferior Frontal Gyrus, Triangular Part (Right) – Superior Frontal Gyrus, Medial Part (Left) | 0.452 | 0.510 | 0.504 | 0.400 | 4.062 | 0.047 |
| Inferior Frontal Gyrus, Triangular Part (Right) – Middle Frontal Gyrus (Right) | 0.149 | 0.206 | 0.183 | 0.114 | 4.004 | 0.049 |
| Superior Temporal Gyrus (Right) – Superior Frontal Gyrus (Right) | 0.259 | 0.357 | 0.268 | 0.265 | 3.872 | 0.052 |
| Precentral Gyrus (Right) – Superior Frontal Gyrus, Medial Part (Left) | 0.275 | 0.362 | 0.350 | 0.299 | 3.833 | 0.054 |
| Superior Frontal Gyrus (Right) – Middle Temporal Gyrus (Left) | 0.274 | 0.330 | 0.348 | 0.276 | 3.738 | 0.057 |
| Superior Temporal Gyrus (Right) – Postcentral Gyrus (Right) | 0.311 | 0.380 | 0.315 | 0.284 | 3.719 | 0.057 |
| Superior Temporal Gyrus (Right) – Middle Temporal Gyrus (Left) | 0.172 | 0.217 | 0.229 | 0.164 | 3.549 | 0.063 |
| Inferior Frontal Gyrus, Opercular Part (Right) – Precentral Gyrus (Left) | 0.200 | 0.291 | 0.206 | 0.190 | 3.403 | 0.069 |
| Middle Temporal Gyrus (Left) – Middle Frontal Gyrus (Left) | 0.351 | 0.416 | 0.395 | 0.336 | 3.389 | 0.069 |
| Superior Frontal Gyrus (Left) – Middle Frontal Gyrus (Left) | 0.306 | 0.352 | 0.356 | 0.276 | 3.341 | 0.071 |
| Inferior Frontal Gyrus, Opercular Part (Right) – Superior Frontal Gyrus, Medial Part (Left) | 0.335 | 0.482 | 0.393 | 0.344 | 3.283 | 0.074 |
| Inferior Frontal Gyrus, Triangular Part (Right) – Inferior Frontal Gyrus, Orbital Part (Left) | 0.327 | 0.455 | 0.376 | 0.382 | 3.240 | 0.075 |
| Inferior Frontal Gyrus, Triangular Part (Right) – Precentral Gyrus (Left) | 0.185 | 0.246 | 0.240 | 0.208 | 3.148 | 0.080 |
| Superior Frontal Gyrus, Medial Part (Right) – Middle Temporal Gyrus (Left) | 0.279 | 0.335 | 0.342 | 0.285 | 3.099 | 0.082 |
| Superior Frontal Gyrus, Medial Part (Right) – Postcentral Gyrus (Left) | 0.256 | 0.347 | 0.312 | 0.294 | 3.059 | 0.084 |
| Inferior Frontal Gyrus, Opercular Part (Right) – Superior Frontal Gyrus, Medial Part (Right) | 0.277 | 0.313 | 0.320 | 0.268 | 3.058 | 0.084 |
| Postcentral Gyrus (Right) – Superior Frontal Gyrus, Medial Part (Left) | 0.395 | 0.463 | 0.416 | 0.388 | 3.030 | 0.085 |
| Superior Frontal Gyrus, Medial Part (Right) – Inferior Frontal Gyrus, Orbital Part (Left) | 0.303 | 0.383 | 0.258 | 0.241 | 2.937 | 0.090 |
| Inferior Frontal Gyrus, Orbital Part (Left) – Postcentral Gyrus (Left) | 0.329 | 0.357 | 0.388 | 0.317 | 2.934 | 0.090 |
| Middle Frontal Gyrus (Right) – Precentral Gyrus (Left) | 0.473 | 0.541 | 0.534 | 0.487 | 2.875 | 0.094 |
| Inferior Frontal Gyrus, Triangular Part (Right) – Supramarginal Gyrus (Left) | 0.196 | 0.236 | 0.243 | 0.195 | 2.807 | 0.098 |
| Postcentral Gyrus (Left) – Precentral Gyrus (Left) | 0.332 | 0.356 | 0.403 | 0.316 | 2.614 | 0.110 |
| Superior Temporal Gyrus (Left) – Middle Temporal Gyrus (Left) | 0.266 | 0.315 | 0.284 | 0.265 | 2.568 | 0.113 |
| Superior Frontal Gyrus (Right) – Postcentral Gyrus (Left) | 0.264 | 0.356 | 0.314 | 0.312 | 2.502 | 0.118 |
| Middle Temporal Gyrus (Left) – Superior Frontal Gyrus (Left) | 0.294 | 0.341 | 0.340 | 0.292 | 2.475 | 0.119 |
| Precentral Gyrus (Right) – Superior Frontal Gyrus (Left) | 0.282 | 0.336 | 0.336 | 0.295 | 2.452 | 0.121 |
| Inferior Frontal Gyrus, Triangular Part (Right) – Postcentral Gyrus (Right) | 0.363 | 0.486 | 0.428 | 0.439 | 2.383 | 0.126 |
| Postcentral Gyrus (Right) – Precentral Gyrus (Left) | 0.583 | 0.676 | 0.643 | 0.619 | 2.370 | 0.128 |
| Superior Frontal Gyrus, Medial Part (Right) – Inferior Frontal Gyrus, Triangular Part (Left) | 0.287 | 0.318 | 0.272 | 0.202 | 2.341 | 0.130 |
| Middle Frontal Gyrus (Right) – Supramarginal Gyrus (Left) | 0.263 | 0.325 | 0.314 | 0.298 | 2.284 | 0.135 |
| Middle Frontal Gyrus (Left) – Superior Frontal Gyrus, Medial Part (Left) | 0.277 | 0.366 | 0.360 | 0.323 | 2.237 | 0.139 |
| Inferior Frontal Gyrus, Opercular Part (Right) – Superior Frontal Gyrus (Right) | 0.317 | 0.369 | 0.345 | 0.316 | 2.227 | 0.139 |
| Inferior Frontal Gyrus, Orbital Part (Left) – Inferior Frontal Gyrus, Triangular Part (Left) | 0.247 | 0.317 | 0.348 | 0.304 | 2.223 | 0.140 |
| Superior Temporal Gyrus (Right) – Middle Frontal Gyrus (Left) | 0.342 | 0.385 | 0.381 | 0.334 | 2.209 | 0.141 |
| Middle Frontal Gyrus (Right) – Middle Frontal Gyrus (Left) | 0.283 | 0.320 | 0.350 | 0.299 | 2.185 | 0.143 |
| Precentral Gyrus (Right) – Inferior Frontal Gyrus, Orbital Part (Left) | 0.476 | 0.556 | 0.540 | 0.513 | 2.162 | 0.145 |
| Precentral Gyrus (Right) – Superior Temporal Gyrus (Left) | 0.232 | 0.280 | 0.266 | 0.230 | 2.103 | 0.151 |
| Middle Frontal Gyrus (Right) – Postcentral Gyrus (Right) | 0.484 | 0.529 | 0.519 | 0.479 | 2.040 | 0.157 |
| Superior Frontal Gyrus (Right) – Inferior Frontal Gyrus, Triangular Part (Left) | 0.282 | 0.331 | 0.356 | 0.314 | 2.014 | 0.160 |
| Middle Frontal Gyrus (Right) – Superior Frontal Gyrus, Medial Part (Left) | 0.377 | 0.426 | 0.414 | 0.391 | 1.998 | 0.161 |
| Superior Temporal Gyrus (Left) – Inferior Frontal Gyrus, Triangular Part (Left) | 0.270 | 0.335 | 0.299 | 0.283 | 1.872 | 0.175 |
| Inferior Frontal Gyrus, Opercular Part (Right) – Inferior Frontal Gyrus, Triangular Part (Right) | 0.354 | 0.398 | 0.376 | 0.335 | 1.862 | 0.176 |
| Superior Frontal Gyrus (Right) – Superior Frontal Gyrus, Medial Part (Left) | 0.295 | 0.358 | 0.374 | 0.348 | 1.857 | 0.177 |
| Middle Frontal Gyrus (Right) – Superior Frontal Gyrus, Medial Part (Right) | 0.365 | 0.397 | 0.382 | 0.349 | 1.844 | 0.178 |
| Superior Temporal Gyrus (Right) – Superior Frontal Gyrus (Left) | 0.427 | 0.470 | 0.446 | 0.420 | 1.798 | 0.184 |
| Precentral Gyrus (Right) – Precentral Gyrus (Left) | 0.246 | 0.336 | 0.270 | 0.283 | 1.726 | 0.193 |
| Superior Frontal Gyrus (Right) – Middle Frontal Gyrus (Left) | 0.286 | 0.361 | 0.291 | 0.279 | 1.699 | 0.196 |
| Superior Frontal Gyrus (Right) – Superior Temporal Gyrus (Left) | 0.317 | 0.382 | 0.331 | 0.309 | 1.673 | 0.199 |
| Middle Temporal Gyrus (Left) – Supramarginal Gyrus (Left) | 0.250 | 0.307 | 0.333 | 0.296 | 1.644 | 0.203 |
| Middle Frontal Gyrus (Right) – Precentral Gyrus (Right) | 0.276 | 0.304 | 0.299 | 0.268 | 1.633 | 0.205 |
| Middle Frontal Gyrus (Right) – Superior Frontal Gyrus (Left) | 0.307 | 0.365 | 0.348 | 0.333 | 1.620 | 0.207 |
| Middle Frontal Gyrus (Left) – Supramarginal Gyrus (Left) | 0.274 | 0.353 | 0.349 | 0.328 | 1.532 | 0.219 |
| Superior Frontal Gyrus (Left) – Inferior Frontal Gyrus, Orbital Part (Left) | 0.246 | 0.332 | 0.294 | 0.300 | 1.523 | 0.221 |
| Precentral Gyrus (Right) – Supramarginal Gyrus (Left) | 0.239 | 0.310 | 0.290 | 0.291 | 1.520 | 0.221 |
| Superior Frontal Gyrus, Medial Part (Right) – Superior Frontal Gyrus, Medial Part (Left) | 0.259 | 0.286 | 0.249 | 0.195 | 1.500 | 0.224 |
| Middle Frontal Gyrus (Left) – Inferior Frontal Gyrus, Triangular Part (Left) | 0.440 | 0.528 | 0.531 | 0.505 | 1.486 | 0.226 |
| Middle Frontal Gyrus (Right) – Postcentral Gyrus (Left) | 0.473 | 0.515 | 0.534 | 0.497 | 1.440 | 0.234 |
| Superior Frontal Gyrus, Medial Part (Right) – Superior Frontal Gyrus (Left) | 0.291 | 0.434 | 0.387 | 0.429 | 1.407 | 0.239 |
| Inferior Frontal Gyrus, Opercular Part (Right) – Superior Temporal Gyrus (Left) | 0.359 | 0.391 | 0.345 | 0.302 | 1.396 | 0.241 |
| Inferior Frontal Gyrus, Triangular Part (Left) – Supramarginal Gyrus (Left) | 0.352 | 0.363 | 0.383 | 0.332 | 1.383 | 0.243 |
| Inferior Frontal Gyrus, Opercular Part (Right) – Postcentral Gyrus (Left) | 0.372 | 0.447 | 0.435 | 0.400 | 1.367 | 0.246 |
| Inferior Frontal Gyrus, Opercular Part (Right) – Middle Frontal Gyrus (Left) | 0.299 | 0.325 | 0.310 | 0.269 | 1.363 | 0.246 |
| Superior Frontal Gyrus, Medial Part (Left) – Supramarginal Gyrus (Left) | 0.360 | 0.405 | 0.426 | 0.381 | 1.361 | 0.247 |
| Superior Frontal Gyrus, Medial Part (Right) – Superior Temporal Gyrus (Left) | 0.505 | 0.537 | 0.543 | 0.505 | 1.287 | 0.260 |
| Superior Temporal Gyrus (Right) – Inferior Frontal Gyrus, Opercular Part (Right) | 0.430 | 0.463 | 0.483 | 0.442 | 1.272 | 0.263 |
| Middle Frontal Gyrus (Left) – Postcentral Gyrus (Left) | 0.420 | 0.473 | 0.461 | 0.431 | 1.260 | 0.265 |
| Precentral Gyrus (Left) – Supramarginal Gyrus (Left) | 0.315 | 0.391 | 0.412 | 0.396 | 1.178 | 0.281 |
| Superior Frontal Gyrus (Right) – Precentral Gyrus (Left) | 0.291 | 0.343 | 0.284 | 0.266 | 1.176 | 0.281 |
| Superior Frontal Gyrus (Left) – Supramarginal Gyrus (Left) | 0.373 | 0.422 | 0.424 | 0.399 | 1.153 | 0.286 |
| Superior Temporal Gyrus (Right) – Superior Frontal Gyrus, Medial Part (Right) | 0.330 | 0.374 | 0.329 | 0.319 | 1.150 | 0.287 |
| Superior Frontal Gyrus (Left) – Precentral Gyrus (Left) | 0.315 | 0.359 | 0.350 | 0.324 | 1.084 | 0.301 |
| Precentral Gyrus (Right) – Middle Frontal Gyrus (Left) | 0.593 | 0.668 | 0.631 | 0.630 | 1.082 | 0.301 |
| Superior Temporal Gyrus (Left) – Precentral Gyrus (Left) | 0.294 | 0.314 | 0.315 | 0.282 | 1.076 | 0.303 |
| Superior Frontal Gyrus, Medial Part (Left) – Precentral Gyrus (Left) | 0.331 | 0.359 | 0.423 | 0.371 | 1.058 | 0.307 |
| Postcentral Gyrus (Left) – Supramarginal Gyrus (Left) | 0.505 | 0.583 | 0.541 | 0.541 | 0.998 | 0.321 |
| Middle Temporal Gyrus (Left) – Postcentral Gyrus (Left) | 0.136 | 0.250 | 0.202 | 0.242 | 0.989 | 0.323 |
| Inferior Frontal Gyrus, Opercular Part (Right) – Postcentral Gyrus (Right) | 0.302 | 0.341 | 0.308 | 0.291 | 0.965 | 0.329 |
| Inferior Frontal Gyrus, Triangular Part (Left) – Precentral Gyrus (Left) | 0.556 | 0.654 | 0.600 | 0.607 | 0.952 | 0.332 |
| Inferior Frontal Gyrus, Opercular Part (Right) – Precentral Gyrus (Right) | 0.299 | 0.376 | 0.281 | 0.292 | 0.899 | 0.346 |
| Inferior Frontal Gyrus, Triangular Part (Right) – Precentral Gyrus (Right) | 0.336 | 0.377 | 0.421 | 0.393 | 0.892 | 0.348 |
| Superior Temporal Gyrus (Left) – Supramarginal Gyrus (Left) | 0.301 | 0.321 | 0.322 | 0.296 | 0.830 | 0.365 |
| Superior Frontal Gyrus, Medial Part (Right) – Precentral Gyrus (Left) | 0.221 | 0.287 | 0.209 | 0.216 | 0.825 | 0.366 |
| Postcentral Gyrus (Right) – Middle Temporal Gyrus (Left) | 0.381 | 0.466 | 0.457 | 0.478 | 0.816 | 0.369 |
| Superior Temporal Gyrus (Right) – Inferior Frontal Gyrus, Triangular Part (Right) | 0.346 | 0.363 | 0.371 | 0.330 | 0.810 | 0.371 |
| Superior Temporal Gyrus (Right) – Superior Frontal Gyrus, Medial Part (Left) | 0.520 | 0.552 | 0.522 | 0.506 | 0.781 | 0.379 |
| Superior Temporal Gyrus (Left) – Postcentral Gyrus (Left) | 0.350 | 0.423 | 0.407 | 0.420 | 0.717 | 0.400 |
| Superior Temporal Gyrus (Right) – Postcentral Gyrus (Left) | 0.676 | 0.723 | 0.714 | 0.696 | 0.712 | 0.401 |
| Middle Temporal Gyrus (Left) – Superior Frontal Gyrus, Medial Part (Left) | 0.140 | 0.273 | 0.182 | 0.250 | 0.672 | 0.415 |
| Inferior Frontal Gyrus, Opercular Part (Right) – Inferior Frontal Gyrus, Orbital Part (Left) | 0.487 | 0.508 | 0.457 | 0.427 | 0.668 | 0.416 |
| Postcentral Gyrus (Right) – Precentral Gyrus (Right) | 0.336 | 0.435 | 0.337 | 0.379 | 0.643 | 0.425 |
| Superior Frontal Gyrus (Left) – Postcentral Gyrus (Left) | 0.215 | 0.325 | 0.321 | 0.369 | 0.617 | 0.434 |
| Inferior Frontal Gyrus, Orbital Part (Left) – Precentral Gyrus (Left) | 0.346 | 0.356 | 0.370 | 0.333 | 0.615 | 0.435 |
| Superior Frontal Gyrus (Right) – Superior Frontal Gyrus (Left) | 0.257 | 0.356 | 0.262 | 0.305 | 0.611 | 0.437 |
| Postcentral Gyrus (Right) – Postcentral Gyrus (Left) | 0.702 | 0.784 | 0.775 | 0.792 | 0.593 | 0.443 |
| Superior Frontal Gyrus (Right) – Inferior Frontal Gyrus, Orbital Part (Left) | 0.308 | 0.389 | 0.326 | 0.364 | 0.584 | 0.447 |
| Inferior Frontal Gyrus, Opercular Part (Right) – Superior Frontal Gyrus (Left) | 0.240 | 0.254 | 0.259 | 0.238 | 0.510 | 0.477 |
| Inferior Frontal Gyrus, Triangular Part (Left) – Superior Frontal Gyrus, Medial Part (Left) | 0.314 | 0.341 | 0.331 | 0.314 | 0.489 | 0.486 |
| Middle Frontal Gyrus (Right) – Superior Frontal Gyrus (Right) | 0.546 | 0.577 | 0.566 | 0.556 | 0.468 | 0.496 |
| Inferior Frontal Gyrus, Triangular Part (Right) – Superior Frontal Gyrus (Left) | 0.293 | 0.322 | 0.323 | 0.317 | 0.414 | 0.522 |
| Superior Frontal Gyrus, Medial Part (Right) – Precentral Gyrus (Right) | 0.312 | 0.368 | 0.327 | 0.343 | 0.391 | 0.533 |
| Inferior Frontal Gyrus, Triangular Part (Right) – Middle Frontal Gyrus (Left) | 0.300 | 0.337 | 0.325 | 0.326 | 0.363 | 0.549 |
| Inferior Frontal Gyrus, Triangular Part (Right) – Superior Temporal Gyrus (Left) | 0.297 | 0.301 | 0.348 | 0.388 | 0.274 | 0.602 |
| Middle Temporal Gyrus (Left) – Inferior Frontal Gyrus, Triangular Part (Left) | 0.242 | 0.275 | 0.261 | 0.259 | 0.265 | 0.608 |
| Inferior Frontal Gyrus, Triangular Part (Right) – Inferior Frontal Gyrus, Triangular Part (Left) | 0.246 | 0.306 | 0.239 | 0.331 | 0.261 | 0.610 |
| Inferior Frontal Gyrus, Orbital Part (Left) – Superior Frontal Gyrus, Medial Part (Left) | 0.352 | 0.334 | 0.317 | 0.323 | 0.223 | 0.638 |
| Middle Frontal Gyrus (Left) – Inferior Frontal Gyrus, Orbital Part (Left) | 0.247 | 0.302 | 0.260 | 0.277 | 0.208 | 0.650 |
| Superior Frontal Gyrus (Left) – Inferior Frontal Gyrus, Triangular Part (Left) | 0.258 | 0.316 | 0.296 | 0.322 | 0.199 | 0.657 |
| Middle Temporal Gyrus (Left) – Precentral Gyrus (Left) | 0.287 | 0.343 | 0.312 | 0.342 | 0.180 | 0.673 |
| Superior Frontal Gyrus (Right) – Postcentral Gyrus (Right) | 0.394 | 0.404 | 0.394 | 0.381 | 0.108 | 0.743 |
| Superior Frontal Gyrus, Medial Part (Right) – Superior Frontal Gyrus (Right) | 0.249 | 0.280 | 0.258 | 0.272 | 0.080 | 0.778 |
| Inferior Frontal Gyrus, Opercular Part (Right) – Middle Frontal Gyrus (Right) | 0.367 | 0.400 | 0.321 | 0.344 | 0.056 | 0.813 |
| Inferior Frontal Gyrus, Triangular Part (Right) – Middle Temporal Gyrus (Left) | 0.291 | 0.341 | 0.291 | 0.330 | 0.048 | 0.827 |
| Middle Frontal Gyrus (Right) – Superior Temporal Gyrus (Left) | 0.257 | 0.251 | 0.231 | 0.233 | 0.022 | 0.881 |
| Superior Frontal Gyrus, Medial Part (Left) – Postcentral Gyrus (Left) | 0.382 | 0.367 | 0.377 | 0.367 | 0.008 | 0.930 |
| Superior Temporal Gyrus (Right) – Middle Frontal Gyrus (Right) | 0.656 | 0.653 | 0.636 | 0.628 | 0.004 | 0.949 |
| Inferior Frontal Gyrus, Opercular Part (Right) – Middle Temporal Gyrus (Left) | 0.404 | 0.385 | 0.347 | 0.326 | 0.002 | 0.961 |

Note: The overall region defined by the edge connections of font shadowing boundaries exhibits statistical significance at pFWE = 0.010.

Abbreviations: FC, functional connectivity; FWE, family-wise error; NBS, network-based statistic; zFC, Fisher z-transformed functional connectivity; Inf, inferior; Mid, middle; Oper, opercular; Orb, orbital; Sup, superior; Tri, triangular; L, left; R, right.

## Supplementary Table 6. Exploratory within-group pre–post changes in individual connections of the significant NBS component.

| **Group** | **Connection** | **Pre_mean_zFC** | **Post_mean_zFC** | **t_value** | **p_raw** |
| --- | --- | --- | --- | --- | --- |
| Active | Inferior Frontal Gyrus, Triangular Part (Right) – Superior Frontal Gyrus, Medial Part (Right) | 0.225 | 0.401 | 3.357 | 0.002 |
|  | Superior Frontal Gyrus, Medial Part (Right) – Supramarginal Gyrus (Left) | 0.491 | 0.666 | 3.191 | 0.003 |
|  | Middle Frontal Gyrus (Left) – Precentral Gyrus (Left) | 0.314 | 0.458 | 3.020 | 0.004 |
|  | Precentral Gyrus (Right) – Postcentral Gyrus (Left) | 0.239 | 0.342 | 2.715 | 0.010 |
|  | Postcentral Gyrus (Right) – Superior Frontal Gyrus (Left) | 0.466 | 0.622 | 2.620 | 0.012 |
|  | Superior Temporal Gyrus (Left) – Inferior Frontal Gyrus, Orbital Part (Left) | 0.257 | 0.367 | 2.578 | 0.014 |
|  | Superior Frontal Gyrus (Right) – Supramarginal Gyrus (Left) | 0.393 | 0.549 | 2.489 | 0.017 |
|  | Inferior Frontal Gyrus, Opercular Part (Right) – Inferior Frontal Gyrus, Triangular Part (Left) | 0.212 | 0.356 | 2.477 | 0.017 |
|  | Superior Temporal Gyrus (Right) – Precentral Gyrus (Left) | 0.459 | 0.587 | 2.303 | 0.026 |
|  | Middle Frontal Gyrus (Right) – Inferior Frontal Gyrus, Orbital Part (Left) | 0.157 | 0.261 | 2.294 | 0.027 |
|  | Superior Frontal Gyrus (Left) – Superior Frontal Gyrus, Medial Part (Left) | 0.173 | 0.306 | 2.175 | 0.035 |
|  | Inferior Frontal Gyrus, Orbital Part (Left) – Supramarginal Gyrus (Left) | 0.384 | 0.480 | 2.020 | 0.050 |
|  | Precentral Gyrus (Right) – Middle Temporal Gyrus (Left) | 0.104 | 0.214 | 2.004 | 0.052 |
|  | Superior Frontal Gyrus, Medial Part (Right) – Middle Frontal Gyrus (Left) | 0.370 | 0.481 | 1.940 | 0.059 |
|  | Middle Frontal Gyrus (Right) – Inferior Frontal Gyrus, Triangular Part (Left) | 0.162 | 0.236 | 1.931 | 0.060 |
|  | Postcentral Gyrus (Right) – Supramarginal Gyrus (Left) | 0.258 | 0.340 | 1.829 | 0.075 |
|  | Inferior Frontal Gyrus, Triangular Part (Right) – Superior Frontal Gyrus (Right) | 0.234 | 0.331 | 1.816 | 0.077 |
|  | Superior Frontal Gyrus, Medial Part (Right) – Postcentral Gyrus (Right) | 0.160 | 0.254 | 1.808 | 0.078 |
|  | Superior Temporal Gyrus (Right) – Inferior Frontal Gyrus, Orbital Part (Left) | 0.179 | 0.272 | 1.726 | 0.092 |
|  | Superior Temporal Gyrus (Right) – Supramarginal Gyrus (Left) | 0.438 | 0.540 | 1.688 | 0.099 |
|  | Postcentral Gyrus (Right) – Inferior Frontal Gyrus, Triangular Part (Left) | 0.148 | 0.222 | 1.610 | 0.115 |
|  | Superior Temporal Gyrus (Left) – Superior Frontal Gyrus (Left) | 0.232 | 0.290 | 1.584 | 0.121 |
|  | Precentral Gyrus (Right) – Inferior Frontal Gyrus, Triangular Part (Left) | 0.242 | 0.322 | 1.561 | 0.126 |
|  | Superior Temporal Gyrus (Right) – Inferior Frontal Gyrus, Triangular Part (Left) | 0.194 | 0.263 | 1.552 | 0.128 |
|  | Middle Temporal Gyrus (Left) – Inferior Frontal Gyrus, Orbital Part (Left) | 0.241 | 0.312 | 1.532 | 0.133 |
|  | Middle Frontal Gyrus (Right) – Middle Temporal Gyrus (Left) | 0.215 | 0.286 | 1.513 | 0.138 |
|  | Superior Temporal Gyrus (Left) – Superior Frontal Gyrus, Medial Part (Left) | 0.326 | 0.410 | 1.484 | 0.145 |
|  | Postcentral Gyrus (Right) – Inferior Frontal Gyrus, Orbital Part (Left) | 0.146 | 0.235 | 1.480 | 0.146 |
|  | Superior Temporal Gyrus (Right) – Superior Temporal Gyrus (Left) | 0.174 | 0.244 | 1.461 | 0.152 |
|  | Superior Frontal Gyrus (Right) – Precentral Gyrus (Right) | 0.334 | 0.402 | 1.446 | 0.156 |
|  | Superior Temporal Gyrus (Left) – Middle Frontal Gyrus (Left) | 0.256 | 0.310 | 1.423 | 0.162 |
|  | Postcentral Gyrus (Right) – Middle Frontal Gyrus (Left) | 0.283 | 0.357 | 1.391 | 0.172 |
|  | Inferior Frontal Gyrus, Opercular Part (Right) – Supramarginal Gyrus (Left) | 0.135 | 0.196 | 1.316 | 0.195 |
|  | Inferior Frontal Gyrus, Triangular Part (Right) – Middle Frontal Gyrus (Right) | 0.149 | 0.206 | 1.198 | 0.238 |
|  | Inferior Frontal Gyrus, Triangular Part (Right) – Postcentral Gyrus (Left) | 0.289 | 0.335 | 1.067 | 0.292 |
|  | Inferior Frontal Gyrus, Triangular Part (Left) – Postcentral Gyrus (Left) | 0.336 | 0.409 | 1.014 | 0.317 |
|  | Superior Temporal Gyrus (Right) – Precentral Gyrus (Right) | 0.311 | 0.356 | 0.936 | 0.355 |
|  | Inferior Frontal Gyrus, Triangular Part (Right) – Superior Frontal Gyrus, Medial Part (Left) | 0.452 | 0.510 | 0.870 | 0.389 |
| Sham | Postcentral Gyrus (Right) – Middle Frontal Gyrus (Left) | 0.393 | 0.278 | -2.623 | 0.012 |
|  | Postcentral Gyrus (Right) – Inferior Frontal Gyrus, Triangular Part (Left) | 0.221 | 0.129 | -2.375 | 0.022 |
|  | Postcentral Gyrus (Right) – Inferior Frontal Gyrus, Orbital Part (Left) | 0.238 | 0.149 | -2.321 | 0.025 |
|  | Inferior Frontal Gyrus, Triangular Part (Right) – Superior Frontal Gyrus, Medial Part (Left) | 0.504 | 0.400 | -2.275 | 0.028 |
|  | Inferior Frontal Gyrus, Triangular Part (Left) – Postcentral Gyrus (Left) | 0.400 | 0.297 | -2.206 | 0.033 |
|  | Precentral Gyrus (Right) – Middle Temporal Gyrus (Left) | 0.194 | 0.098 | -2.198 | 0.033 |
|  | Precentral Gyrus (Right) – Inferior Frontal Gyrus, Triangular Part (Left) | 0.281 | 0.176 | -2.156 | 0.037 |
|  | Superior Temporal Gyrus (Left) – Superior Frontal Gyrus, Medial Part (Left) | 0.396 | 0.300 | -2.154 | 0.037 |
|  | Inferior Frontal Gyrus, Opercular Part (Right) – Supramarginal Gyrus (Left) | 0.200 | 0.110 | -2.110 | 0.041 |
|  | Middle Temporal Gyrus (Left) – Inferior Frontal Gyrus, Orbital Part (Left) | 0.319 | 0.239 | -2.082 | 0.043 |
|  | Inferior Frontal Gyrus, Triangular Part (Right) – Postcentral Gyrus (Left) | 0.349 | 0.282 | -2.074 | 0.044 |
|  | Superior Temporal Gyrus (Right) – Precentral Gyrus (Right) | 0.395 | 0.315 | -2.040 | 0.048 |
|  | Superior Temporal Gyrus (Left) – Middle Frontal Gyrus (Left) | 0.315 | 0.255 | -1.985 | 0.054 |
|  | Middle Frontal Gyrus (Right) – Middle Temporal Gyrus (Left) | 0.283 | 0.202 | -1.926 | 0.061 |
|  | Postcentral Gyrus (Right) – Supramarginal Gyrus (Left) | 0.316 | 0.245 | -1.781 | 0.082 |
|  | Inferior Frontal Gyrus, Orbital Part (Left) – Supramarginal Gyrus (Left) | 0.453 | 0.383 | -1.751 | 0.087 |
|  | Superior Frontal Gyrus, Medial Part (Right) – Postcentral Gyrus (Right) | 0.215 | 0.143 | -1.746 | 0.088 |
|  | Superior Temporal Gyrus (Right) – Superior Temporal Gyrus (Left) | 0.227 | 0.170 | -1.684 | 0.100 |
|  | Inferior Frontal Gyrus, Triangular Part (Right) – Middle Frontal Gyrus (Right) | 0.183 | 0.114 | -1.666 | 0.103 |
|  | Middle Frontal Gyrus (Right) – Inferior Frontal Gyrus, Triangular Part (Left) | 0.233 | 0.169 | -1.573 | 0.123 |
|  | Superior Frontal Gyrus (Right) – Precentral Gyrus (Right) | 0.357 | 0.289 | -1.522 | 0.136 |
|  | Superior Temporal Gyrus (Right) – Inferior Frontal Gyrus, Triangular Part (Left) | 0.226 | 0.166 | -1.465 | 0.150 |
|  | Superior Temporal Gyrus (Left) – Superior Frontal Gyrus (Left) | 0.291 | 0.243 | -1.438 | 0.158 |
|  | Superior Frontal Gyrus, Medial Part (Right) – Supramarginal Gyrus (Left) | 0.642 | 0.540 | -1.411 | 0.166 |
|  | Superior Temporal Gyrus (Right) – Inferior Frontal Gyrus, Orbital Part (Left) | 0.231 | 0.189 | -1.362 | 0.180 |
|  | Inferior Frontal Gyrus, Triangular Part (Right) – Superior Frontal Gyrus (Right) | 0.237 | 0.181 | -1.295 | 0.203 |
|  | Superior Temporal Gyrus (Right) – Precentral Gyrus (Left) | 0.500 | 0.454 | -1.188 | 0.242 |
|  | Superior Temporal Gyrus (Right) – Supramarginal Gyrus (Left) | 0.467 | 0.407 | -1.176 | 0.246 |
|  | Inferior Frontal Gyrus, Opercular Part (Right) – Inferior Frontal Gyrus, Triangular Part (Left) | 0.279 | 0.225 | -1.135 | 0.263 |
|  | Inferior Frontal Gyrus, Triangular Part (Right) – Superior Frontal Gyrus, Medial Part (Right) | 0.285 | 0.230 | -1.063 | 0.294 |
|  | Superior Frontal Gyrus (Left) – Superior Frontal Gyrus, Medial Part (Left) | 0.232 | 0.176 | -0.980 | 0.333 |
|  | Superior Frontal Gyrus, Medial Part (Right) – Middle Frontal Gyrus (Left) | 0.427 | 0.381 | -0.888 | 0.379 |
|  | Superior Temporal Gyrus (Left) – Inferior Frontal Gyrus, Orbital Part (Left) | 0.325 | 0.297 | -0.857 | 0.397 |
|  | Superior Frontal Gyrus (Right) – Supramarginal Gyrus (Left) | 0.330 | 0.286 | -0.792 | 0.433 |
|  | Middle Frontal Gyrus (Left) – Precentral Gyrus (Left) | 0.408 | 0.377 | -0.770 | 0.446 |
|  | Middle Frontal Gyrus (Right) – Inferior Frontal Gyrus, Orbital Part (Left) | 0.228 | 0.204 | -0.750 | 0.457 |
|  | Precentral Gyrus (Right) – Postcentral Gyrus (Left) | 0.300 | 0.281 | -0.519 | 0.607 |
|  | Postcentral Gyrus (Right) – Superior Frontal Gyrus (Left) | 0.406 | 0.393 | -0.222 | 0.825 |

Abbreviations: FC, functional connectivity; NBS, network-based statistic; zFC, Fisher z-transformed functional connectivity; Inf, inferior; Mid, middle; Oper, opercular; Orb, orbital; Sup, superior; Tri, triangular; L, left; R, right.

## Supplementary Table 7. Associations between NBS-derived mean ΔzFC and cognitive score changes.

| Group | Cognitive outcome | Spearman_rho | *p_raw* | *p_FDR* |
| --- | --- | --- | --- | --- |
| Active | ΔAttention | 0.277 | 0.076 | 0.131 |
|  | ΔLanguage | 0.396 | 0.009 | 0.032 |
|  | ΔMemory | 0.162 | 0.304 | 0.365 |
|  | ΔPraxis | 0.391 | 0.011 | 0.032 |
|  | ΔADAS-Cog total | 0.435 | 0.004 | 0.024 |
|  | ΔMMSE total | -0.456 | 0.002 | 0.024 |
| Sham | ΔAttention | 0.159 | 0.309 | 0.365 |
|  | ΔLanguage | 0.264 | 0.087 | 0.131 |
|  | ΔMemory | 0.151 | 0.334 | 0.365 |
|  | ΔPraxis | -0.003 | 0.987 | 0.987 |
|  | ΔADAS-Cog total | 0.273 | 0.076 | 0.131 |
|  | ΔMMSE total | -0.309 | 0.044 | 0.105 |

Abbreviations: ADAS-Cog, Alzheimer’s Disease Assessment Scale–Cognitive Subscale; FDR, false discovery rate; FC, functional connectivity; MMSE, Mini-Mental State Examination; NBS, network-based statistic; ρ, Spearman correlation coefficient; zFC, Fisher z-transformed functional connectivity; Δ, Week 4 minus baseline.

## Supplementary Table 8. Adverse events and tolerability during 40-Hz tACS

| Variable | Participants who experienced each adverse reaction, No. (%)a | | |
| --- | --- | --- | --- |
|  | Active group (n=42) | Sham group (n = 43) | Grade (CTCAE v5.0) |
| Tinnitus | 3 (7.14%) | 2 (4.65%) | Grade 1 |
| Headache | 2 (4.76%) | 3 (6.98%) | Grade 1 |
| Nausea | 0 (0.0%) | 1 (2.32%) | Grade 1 |
| Dizziness | 5 (11.90%) | 3 (6.98%) | Grade 1 |
| Fatigue | 6 (14.28%) | 5 (11.63%) | Grade 1 |
| Somnolence | 5 (11.90%) | 2 (4.65%) | Grade 1 |
| Pruritus | 8 (19.04%) | 7 (16.28%) | Grade 1 |
| Scalp swelling | 6 (14.28%) | 4 (9.30%) | Grade 1 |
| Cutaneous tingling | 3 (7.14%) | 1 (2.32%) | Grade 1 |
| Cutaneous burning sensation | 4 (9.52%) | 1 (2.32%) | Grade 1 |
| Hypoesthesia | 0 (0.0%) | 0 (0.0%) | None |

Data are presented as the number and percentage of participants reporting each adverse event. Participants could report more than one adverse event. According to the Common Terminology Criteria for Adverse Events, version 5.0 (CTCAE v5.0), all reported adverse events (AEs) were Grade 1 (mild). No Grade 2 or higher adverse events or serious adverse events (SAEs) occurred during the study. The most frequently reported adverse events were pruritus (active group, 19.04%; sham group, 16.28%), fatigue (active group, 14.28%; sham group, 11.63%), and scalp swelling (active group, 14.28%; sham group, 9.30%).

Abbreviations: AE, adverse event; CTCAE, Common Terminology Criteria for Adverse Events; SAE, serious adverse event; tACS, transcranial alternating current stimulation.

# Supplementary figures

## ****Figure S1. Functional connectivity changes and the significant NBS component.****

**(A)** Mean (Δ zFC) matrix in the active group. **(B)** Mean (Δ zFC) matrix in the sham group. For panels A and B, Δ zFC was calculated as Week 4 minus baseline across all ROI pairs. Warm colors indicate increased connectivity, whereas cool colors indicate decreased connectivity. Within-group changes were assessed using paired-samples (t) tests. **(C)** Matrix representation of the significant Group × Time interaction network identified by NBS. Colored cells represent the 38 connections forming the significant component, and cell color indicates the edge-wise ANOVA (F) statistic. Red asterisks mark edges included in the NBS component. Colored bars indicate frontal, temporal, and sensorimotor–parietal modules. Component-level significance was determined using 5000 permutations (pFWE=0.010).

**Abbreviations:** ANOVA, analysis of variance; FC, functional connectivity; FWE, family-wise error; NBS, network-based statistic; ROI, region of interest; (zFC), Fisher (z)-transformed functional connectivity.

## Figure S2. Exploratory within-group functional connectivity changes within the significant NBS component

**(A)** Brain visualization of within-group pre–post connectivity changes in the active group. **(B)** Brain visualization of within-group pre–post connectivity changes in the sham group. **(C)** Circular network representation of within-group functional connectivity (FC) changes in the active group. **(D)** Circular network representation of within-group functional connectivity (FC) changes in the sham group. Only edges with an uncorrected for multiple comparisons paired-samples (t)-test (p<0.05) are displayed. Node color indicates anatomical module, and node size represents node degree within the displayed network. Edge width represents the absolute (t) statistic. Edge color represents the direction of ΔzFC according to a common diverging color scale: red indicates an increase from baseline to Week 4, whereas blue indicates a decrease.

Abbreviations: FC, functional connectivity; NBS, network-based statistic; ROI, region of interest; zFC, Fisher z-transformed functional connectivity; ΔzFC, Week 4 zFC minus baseline zFC.

# Reference

[1]	Y. Huang, L. Shen, J. Huang, X. Xu, Y. Wang, H. Jin, “Efficacy and Safety of tDCS and tACS in Treatment of Major Depressive Disorder: A Randomized, Double-Blind, Factorial Placebo-Controlled Study Design.” *Neuropsychiatr Dis Treat* **2021**, *17*, <https://doi.org/10.2147/ndt.S295945>.

[2]	D. T. Leffa, E. H. Grevet, C. H. D. Bau, M. Schneider, C. P. Ferrazza, R. F. da Silva, M. S. Miranda, F. Picon, S. P. Teche, P. Sanches, D. Pereira, K. Rubia, A. R. Brunoni, J. A. Camprodon, W. Caumo, L. A. Rohde, “Transcranial Direct Current Stimulation vs Sham for the Treatment of Inattention in Adults With Attention-Deficit/Hyperactivity Disorder: The TUNED Randomized Clinical Trial.” *JAMA Psychiatry* **2022**, *79* (9), <https://doi.org/10.1001/jamapsychiatry.2022.2055>.

[3]	T. Wang, S. Yan, Y. Shan, H. Xue, Y. Xing, S. Bi, Z. Chen, H. Xi, Z. Qi, Y. Tang, J. Lu, “Modulation of Cortical and Hippocampal Functional MRI Connectivity Following Transcranial Alternating Current Stimulation in Mild Alzheimer Disease.” *Radiology* **2025**, *315* (3), <https://doi.org/10.1148/radiol.241463>.

[4]	C. S. Sergiou, E. Santarnecchi, S. M. Romanella, M. J. Wieser, I. H. A. Franken, E. G. C. Rassin, J. D. M. van Dongen, “Transcranial Direct Current Stimulation Targeting the Ventromedial Prefrontal Cortex Reduces Reactive Aggression and Modulates Electrophysiological Responses in a Forensic Population.” *Biol Psychiatry Cogn Neurosci Neuroimaging* **2022**, *7* (1), <https://doi.org/10.1016/j.bpsc.2021.05.007>.

[5]	K. Fricke, A. A. Seeber, N. Thirugnanasambandam, W. Paulus, M. A. Nitsche, J. C. Rothwell, “Time course of the induction of homeostatic plasticity generated by repeated transcranial direct current stimulation of the human motor cortex.” *J Neurophysiol* **2011**, *105* (3), <https://doi.org/10.1152/jn.00608.2009>.

[6]	K. Monte-Silva, M. F. Kuo, D. Liebetanz, W. Paulus, M. A. Nitsche, “Shaping the optimal repetition interval for cathodal transcranial direct current stimulation (tDCS).” *J Neurophysiol* **2010**, *103* (4), <https://doi.org/10.1152/jn.00924.2009>.

[7]	E. L. Urquhart, X. Wang, H. Liu, P. J. Fadel, G. Alexandrakis, “Differences in Net Information Flow and Dynamic Connectivity Metrics Between Physically Active and Inactive Subjects Measured by Functional Near-Infrared Spectroscopy (fNIRS) During a Fatiguing Handgrip Task.” *Front Neurosci* **2020**, *14*, <https://doi.org/10.3389/fnins.2020.00167>.

[8]	National Institutes of Health, U.S. Department of Health and Human Services, National Cancer Institute, Common Terminology Criteria for Adverse Events (CTCAE) Version 5.0., <https://pmc.ncbi.nlm.nih.gov/articles/instance/11631963/bin/41392_2024_2048_MOESM5_ESM.pdf#25#1>, accessed.
